# Circular-economy residual-value assessment of retired lithium-ion batteries via degradation prediction and game-theoretic subsidies

**DOI:** 10.1016/j.isci.2026.117430

**Published:** 2026-09-03

**Authors:** Guohui Lan, Yashu Chen, Deli Yao, Jianmin Wang

**Affiliations:** 1 School of Economics and Management, Anhui University of Science and Technology, Huainan, Anhui 232001, China; 2 School of Technology and Business, Anhui University of Science and Technology, Huainan, Anhui 232001, China

**Keywords:** circular economy, retired lithium-ion batteries, residual-value assessment, capacity-degradation prediction, game-theoretic subsidy allocation

## Abstract

Large-scale retirement of lithium-ion batteries enables second-life use in decentralized energy communities, yet capacity-degradation prediction, residual-value assessment, and subsidy allocation are treated in isolation. This study proposes an integrated circular-economy framework linking battery-state prediction with community-level incentive coordination. A physics-constrained transfer-learning hybrid model estimates capacity trajectories and remaining useful life, achieving a root-mean-square error (RMSE) of 0.97%, R^2^ of 0.960, and cross-chemistry transfer RMSE of 1.05%. A dynamic residual-value mapping function and operational degradation-burden index characterize health losses. A Stackelberg leader-follower game is formulated and transformed into single-level optimization via KKT (Karush-Kuhn-Tucker) reformulation, with a dynamic subsidy-allocation mechanism redistributing equilibrium subsidies by degradation contribution, participation duration, and circular-economy factors. Experiments on 240 battery aging samples and a 60-prosumer community show the proposed mechanism yields welfare of 3,610 CNY/day, outperforming fixed uniform allocation under varying subsidy intensities and perturbations.

## Highlights

• A closed-loop framework links battery prediction, valuation, and incentives
• A physics-constrained hybrid deep learning model predicts capacity and life
• Dynamic value mapping connects battery health with community game decisions
• Contribution-weighted subsidies improve welfare, fairness, and robustness

## Graphical abstract

**Figure:**
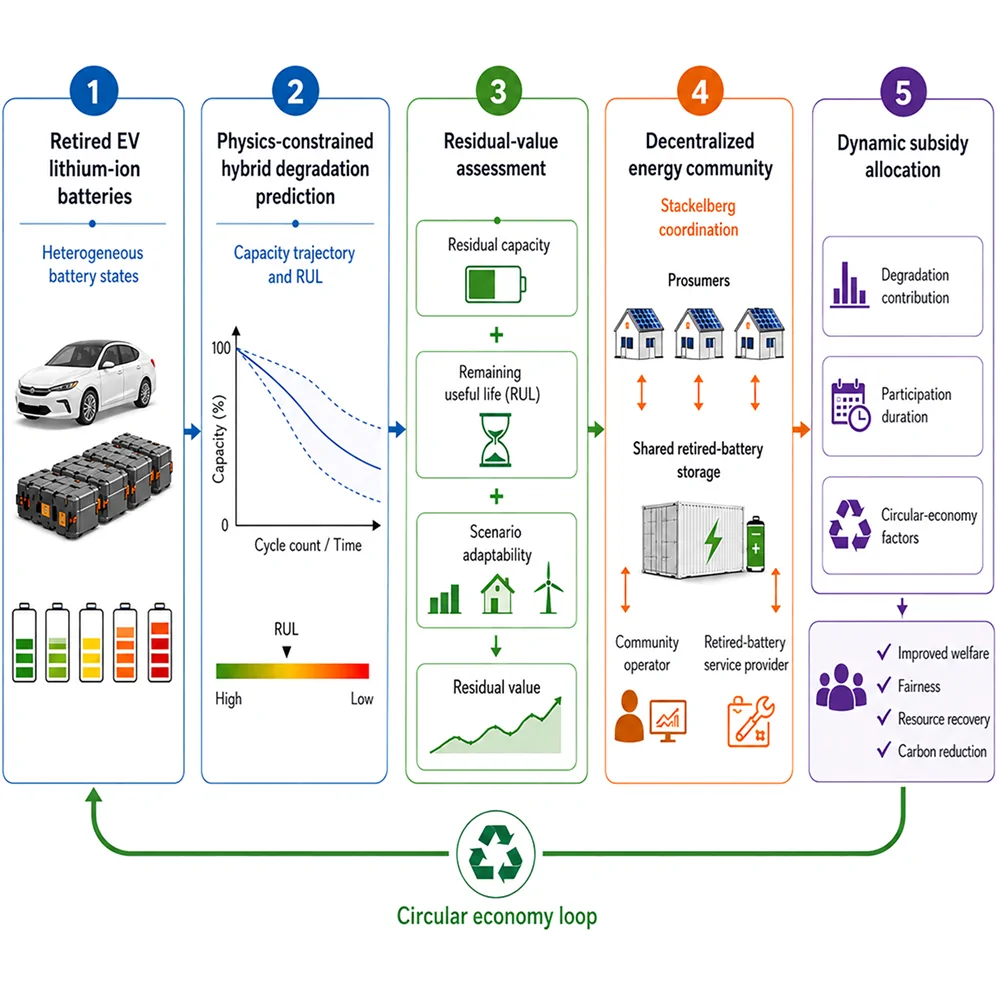

## Teaser

Energy policy; Machine learning; Economics

## Introduction

The global electric-vehicle industry is expanding rapidly, and large-scale decommissioning of power lithium-ion batteries is approaching—a stock regarded by industry as a new round of “green mineral resources.”^1^ When passenger-car power-battery capacity decays to 70%–80% of its rated value, vehicle service life is deemed terminated, yet residual usable capacity still exceeds 30%, carrying significant second-life value.^2^ Over the next decade, cumulative retired lithium-ion batteries in China and worldwide are expected to reach the million-ton scale, with corresponding capacity reaching the hundred-gigawatt-hour scale. How this massive retired-asset stock is managed directly determines whether the full electrification life cycle constitutes a green asset or an environmental burden. Recent studies show that the circular-economy route for retired lithium-ion batteries has shifted from direct material recovery to scenario-oriented second-life utilization.^3^ After disassembly, safety assessment, screening, re-packing, and battery-management-system integration, retired batteries can be reconfigured for stationary battery energy-storage systems.^3^ They have also been deployed in local and community energy systems for photovoltaic self-consumption improvement, load shifting, peak shaving, backup power, and ancillary-service support,^4^ as well as in communication base-station backup power, a typical low-power, low-cycle second-life scenario.^5^ These applications nevertheless face challenges related to heterogeneous aging states, module inconsistency, safety monitoring, residual-life uncertainty, and economic feasibility.^6^ Retired-battery reuse is therefore not merely a capacity-reuse problem but also a scenario-adaptability and residual-value-coordination problem.

In decentralized energy communities, two issues are particularly critical. First, nonlinear and individualized degradation of retired batteries makes residual value difficult to price transparently. Second, the interests of prosumers, energy-storage service providers, and community operators may be misaligned; improper subsidy allocation can induce free-riding or insufficient participation willingness.

Existing research on residual-value evaluation has progressed along three relatively independent technical paths. The technical and economic valuation model quantifies secondary-life fair value through module-level aging models coupled with echelon-scenario income parameterization.^7^ Hybrid prediction of machine learning and physical mechanisms uses early-cycle data to couple electrochemical attenuation mechanisms, achieving capacity-trajectory projection.^8^ Some studies also employ multi-LSTM (long short-term memory) frameworks to achieve interpretable state-of-health estimation based on the cross-scale characteristics of relaxation voltage.^9^ The uncertainty boundary of capacity estimation is deduced from multi-year household energy-storage measured data for field-measurement paths oriented to real operational scenarios.^10^ Each of these three paths has made progress, yet residual-value assessment outputs often remain at the single-cell or module level, decoupled from downstream application scenarios—the residual value is a calculated value, not a used price.

On the side of decentralized energy communities, game theory has been widely used to describe strategic interactions between producers and consumers, operators, and communities. Stackelberg games model pricing-sharing and benefit distribution between energy-storage service providers and multiple users under demand-based electricity tariffs.^11^ The pricing mechanism of shared energy-storage services is further integrated into demand-side complementary regulation, yielding increasingly refined game structures.^12^ Evolutionary games embed carbon pricing, subsidies, and green cards into electricity-market strategic dynamics, depicting long-term multi-agent evolution paths.^13^ It can be seen that echelon-battery capacity-attenuation prediction results are abundant, and game-theoretic research in energy communities continues to deepen, yet the two are rarely connected: attenuation-prediction outputs do not directly enter game payoff functions, and game-subsidy design does not treat remaining battery life, attenuation rate, and participation duration as endogenous variables. This rupture between the residual-value and game-theoretic research threads renders subsidy policy insensitive to residual value, and the capital flow and value flow within the circular-economy closed loop lack an alignment mechanism.

The residual-value evaluation of retired lithium batteries is the key link transforming “surplus capacity” into “market price.” The technical and economic valuation method uses module-level aging models to couple electricity price, cycle times, and retirement recovery value to generate net-present-value or fair-market-price predictions, serving as a paradigm tool in this direction.^14^ The limitation of this paradigm is that module-level degradation behavior is often simplified to a scalar parameter, while internal pack inconsistency, temperature-gradient effects, and operating-system influences are absorbed into residual terms, making estimation accuracy highly sensitive to historical operational data. Life-cycle assessment paths couple economic and environmental objectives through process-level models, embed recycling routes and echelon-utilization scenarios into decision trees, and obtain optimal residual-value differentiation.^15^ Such methods are persuasive at the system level, yet most models depict path selection with static parameters that cannot respond to dynamic value fluctuations in operational scenarios, flattening the temporal “aging” of residual value. An ecosystem perspective further reveals the institutional dependence of residual-value realization, noting that closed-loop value chains require collaborative governance among original-equipment manufacturers, recyclers, and energy-storage service providers and that isolated technology paths are difficult to sustain.^16^ Overall, the current situation is that “we can calculate the number, but we cannot get the price”: assessment outputs mostly remain at the single-unit or module level, decoupled from downstream strategic games and subsidy mechanisms.

Capacity attenuation is the physical basis of residual value, and prediction accuracy directly determines pricing reliability. Generative-learning approaches synthesize training samples under randomized retirement conditions, significantly alleviating data scarcity in state-of-health estimation and demonstrating robustness in recycling-oriented sustainable scenarios.^17^ Deep generative transfer learning aligns the voltage response of heterogeneous second-life batteries across state-of-charge ranges, delivering residual-capacity predictions in an “immediately estimable” manner so that data streams can be acquired on-site.^18^ Physics-informed non-destructive decay-mode decoupling further couples electrochemical mechanisms with early-cycle characteristics, realizing high-fidelity projection from short-term data to long-term trajectories and alleviating the extrapolation risk of purely data-driven models.^19^ Physics-informed neural networks embed electrochemical attenuation mechanisms into the loss function to obtain stable and interpretable degradation trajectories.^20^ Technical reviews emphasize that echelon utilization should follow a “test-screen-recombine-redeploy” process, and capacity prediction must provide reliable consistency indicators during screening and recombination; otherwise, module-level performance will be dragged down by the worst cell.^21^ Cross-domain state-of-health and remaining-useful-life prediction has attracted increasing attention because degradation data collected from different battery chemistries, aging protocols, operating temperatures, and charge-discharge profiles often follow different distributions.^22^ Recent transfer-learning and domain adaptation studies show that models trained on one battery domain suffer performance degradation when directly applied to another domain, whereas transferable attention networks, transfer-adaptive digital-twin frameworks, and domain-adaptive Transformer architectures can improve prediction under limited target-domain data.^23^^,^^24^ These studies provide theoretical support for introducing transfer learning into retired-battery degradation prediction, especially when heterogeneous retired batteries from different chemistries must be evaluated within a unified residual-value assessment framework. Although these works have achieved breakthroughs in prediction accuracy, transferability, and mechanism consistency, their outputs—remaining useful life, state-of-health values, and capacity trajectories—remain “end indicators” that are not explicitly written into downstream residual-value mapping functions nor coupled with the payoff functions of community-operation games.

Decentralized energy communities constitute an important landing scenario for echelon utilization of retired batteries. Stackelberg games model the pricing-response interaction between community operators and multiple users under shared energy storage, with equilibrium solutions accounting for operator profits and user costs.^25^ Bi-level optimization of community energy storage and prosumers further depicts the profit-flexibility trade-off of community energy-storage subjects under upstream power-grid constraints, verifying the existence and feasibility of Stackelberg equilibrium.^26^ Multi-microgrid shared-energy-storage research brings cross-network collaboration into the game perspective, demonstrating local absorption rate and social-welfare improvements when storage resources are shared across borders.^27^ Distributed-game perspectives emphasize Nash-equilibrium solutions under minimum information sharing, embedding games into real-time scheduling through rolling-horizon control to balance privacy and economy.^28^ Reviews of electric-vehicle and smart-grid integration comb the strategic interactions of vehicle-to-grid, shared energy storage, demand response, and other multi-chain configurations, placing game theory as a coordination tool within a macro framework.^29^ Collaborative research combining robust predictive control and game theory integrates uncertainty modeling with strategy equilibrium to adapt to communities with high renewable-energy penetration.^30^ Optimal scheduling of multiple energy stores from long-term and short-term coordination perspectives has also been shown to significantly improve integrated energy-system efficiency.^31^ These studies constitute a relatively mature pedigree of game-theoretic intervention in energy communities, yet two key weaknesses remain. First, the “energy-storage assets” in game payoff functions are mostly abstracted as homogeneous equipment, failing to depict the essential characteristics of heterogeneous capacity, accelerated degradation, and shortened remaining life inherent to retired batteries. Second, subsidies are often introduced as exogenous signals or fixed parameters, with almost no endogenous linkage between subsidy sensitivity, battery remaining life, and degradation contribution. Consequently, although the game structure is refined, the engineering-economic characteristics of retired batteries are not incorporated into strategic variables, and subsidy efficiency is decoupled from battery value extraction.

From the circular-economy perspective, the closed-loop value flow of resources, products, and renewable resources relies on subsidy-allocation mechanisms as the key capital valve.^32^ Research on the circular-economy path of retired power batteries notes that a three-stage pattern of echelon utilization, remanufacturing, and material recycling has basically taken shape yet lacks quantitative value-distribution rules.^33^ Reviews of second-life batteries in fixed energy-storage scenarios indicate that standardized business models and fair pricing mechanisms remain the biggest bottlenecks in industrial implementation; if subsidy policy does not anchor residual value, distortion easily occurs. Stackelberg-game analyses of government-producer-recycler policy transmission verify the significant impact of subsidies on recovery rates and profit distribution, also noting that subsidy-scheme differences negatively affect supply-chain equilibrium.^34^ Blockchain-enabled closed-loop supply-chain coordination further incorporates traceability into the subsidy game, revealing the coupling between information transparency and subsidy efficiency and providing mechanism-design references for subsidy implementation in distributed scenarios.^35^ Overall, existing subsidy-allocation studies mainly emphasize aggregate policy incentives and macro-level effect evaluation, while paying insufficient attention to value-sensitive incentive design in micro-level decentralized energy-community applications. In particular, subsidy weights are rarely linked to the operational degradation contribution of retired batteries, and circular-economy-oriented factors—resource-recovery and conservation contribution, carbon-emission-reduction contribution, and community-stability contribution—are seldom structured as computable allocation weights. As a result, subsidy mechanisms remain weakly connected to retired-battery value realization and participation fairness.

Collectively, capacity-degradation prediction, residual-value assessment, community game modeling, and subsidy allocation have advanced independently, but a structural rupture persists among them. The outputs of degradation prediction do not directly enter game payoff functions; game subsidy design does not treat remaining battery life, degradation rate, and participation duration as endogenous variables; residual-value assessment stays at the calculated-value level without converting into a used price; and subsidy allocation is not tied to battery operational degradation contribution or circular-economy factors. This rupture renders subsidy policy insensitive to residual value, and the capital flow and value flow within the circular-economy closed loop lack an alignment mechanism.

To bridge these gaps, this study develops an integrated framework for retired-battery residual-value assessment and incentive coordination in decentralized energy communities under a circular-economy perspective. The main contributions are 3-fold. (1) A dynamic residual-value assessment mechanism is established by coupling capacity-degradation prediction with residual-value mapping. A physics-constrained transfer-learning hybrid prediction model is developed to estimate the capacity trajectory and remaining useful life of retired batteries under heterogeneous operating conditions; these prediction outputs are then embedded into the residual-value mapping function, enabling residual value to evolve dynamically with battery operating states rather than being treated as a static exogenous parameter. (2) A three-agent Stackelberg leader-follower game is formulated for decentralized energy communities. The community operator and the retired-battery service provider jointly constitute the upper level leader coalition and determine pricing and subsidy signals, while prosumers act as lower level followers and respond through energy-consumption and storage-participation decisions. The dynamic residual-value signal and the operation-induced battery-degradation burden are explicitly incorporated into the payoff structure, thereby linking retired-battery characteristics with strategic community operation. (3) A dynamic subsidy-allocation mechanism is proposed to redistribute equilibrium subsidies according to prosumer-level degradation contribution, participation duration, and circular-economy-oriented correction factors. Resource-recovery and conservation contribution, carbon-emission-reduction contribution, and community-stability contribution are transformed into computable allocation weights, replacing uniform subsidy distribution with a more targeted and sustainability-oriented incentive rule. Overall, the proposed framework forms a closed-loop transmission chain of prediction, valuation, game interaction, and incentive allocation, enabling improvements in capacity-degradation prediction to propagate to residual-value assessment, game-theoretic payoff modeling, and subsidy-allocation fairness.

## Results

### Overall prediction accuracy

**Figure 1:**
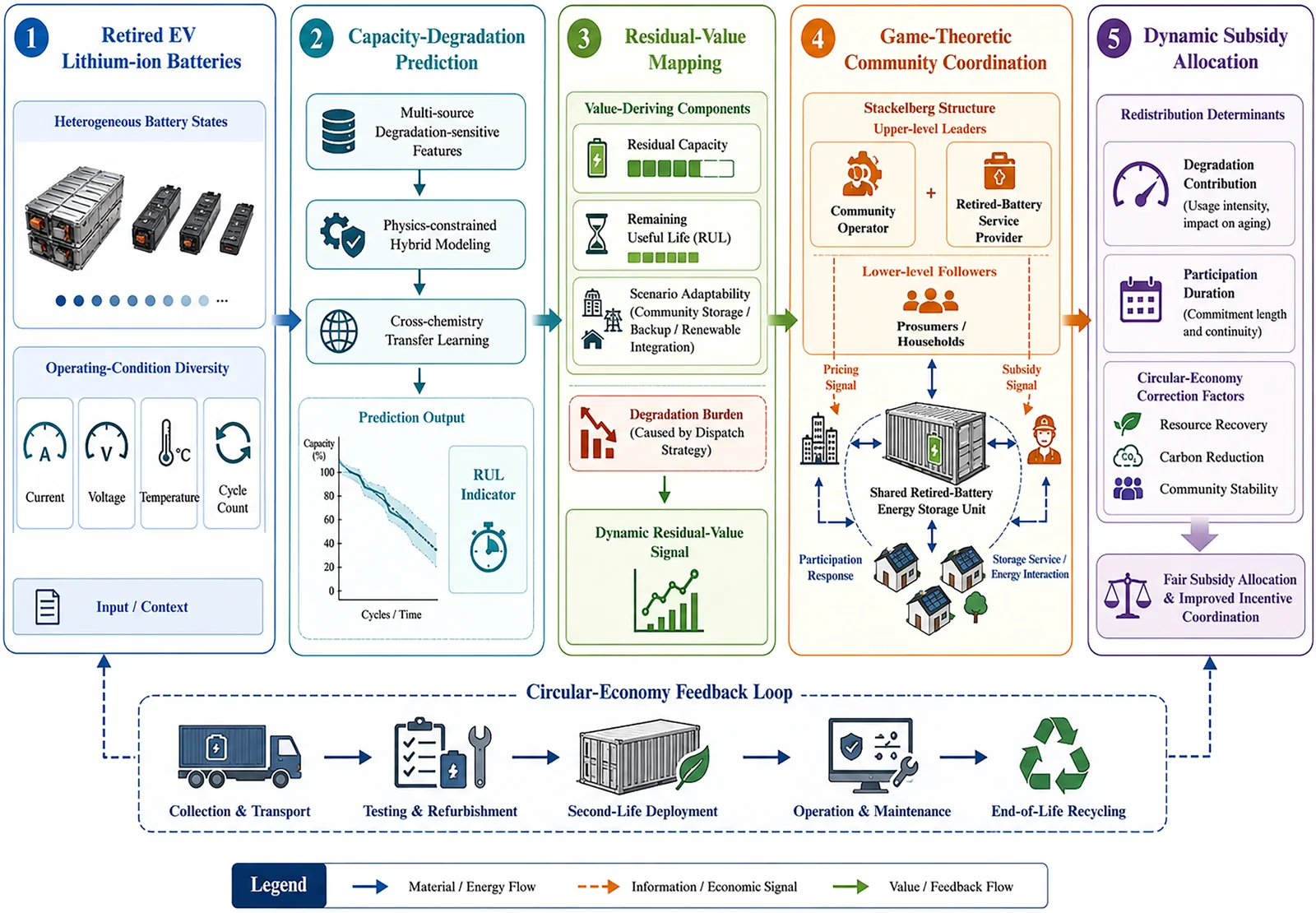
Overall framework for circular-economy residual-value assessment and game-theoretic subsidy allocation of retired lithium-ion batteries The framework links capacity-degradation prediction, residual-value mapping, Stackelberg game coordination, and dynamic subsidy allocation. Battery-state and operational information are transmitted across the four modules to support contribution-sensitive incentive allocation in decentralized energy communities.

**Figure 2:**
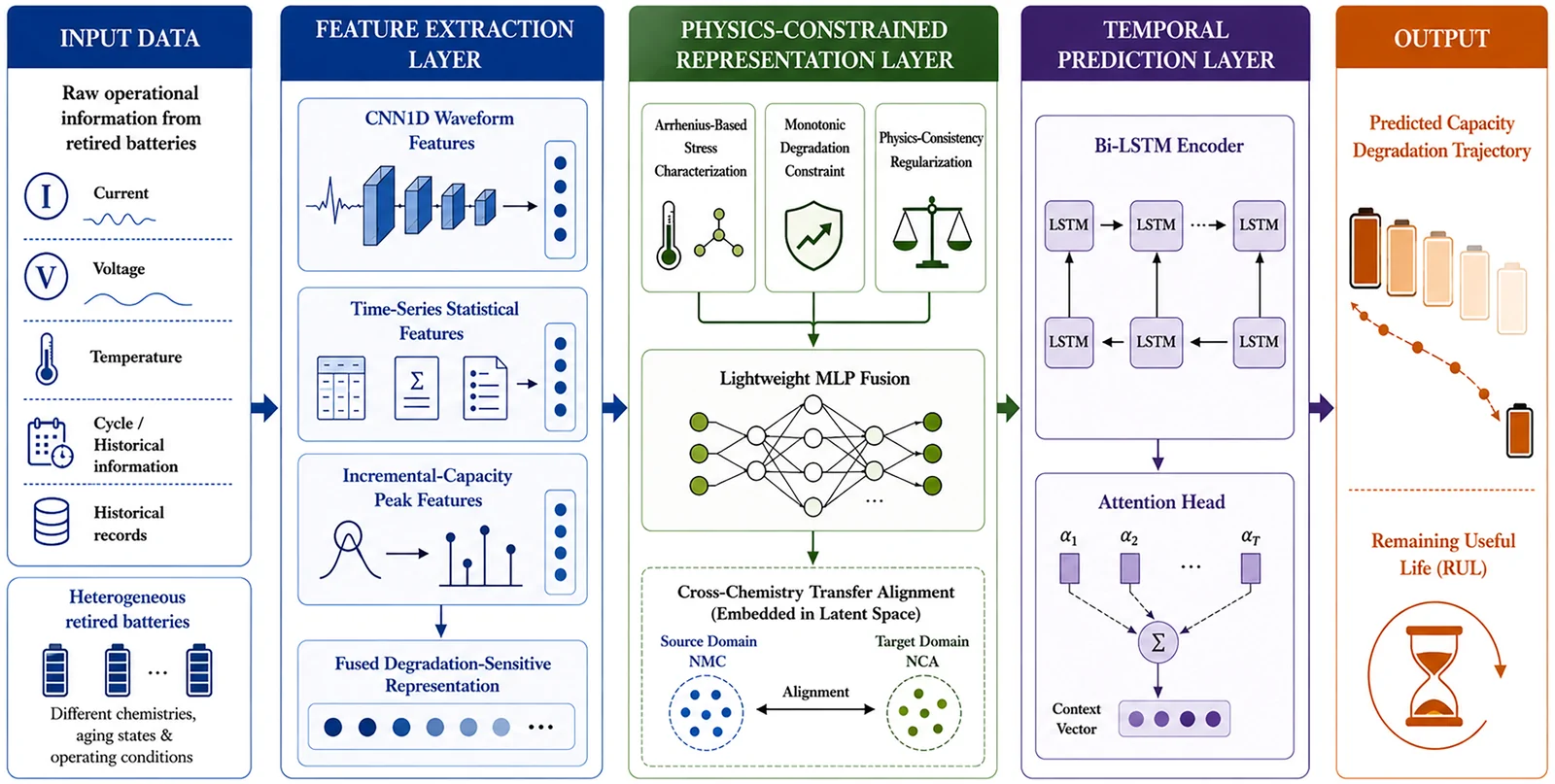
Architecture of the capacity-degradation prediction model The model integrates degradation-sensitive feature extraction, physics-constrained latent representation, cross-chemistry feature alignment, and Bi-LSTM-attention temporal prediction. It outputs the future capacity trajectory and remaining useful life of retired lithium-ion batteries.

**Figure 3:**
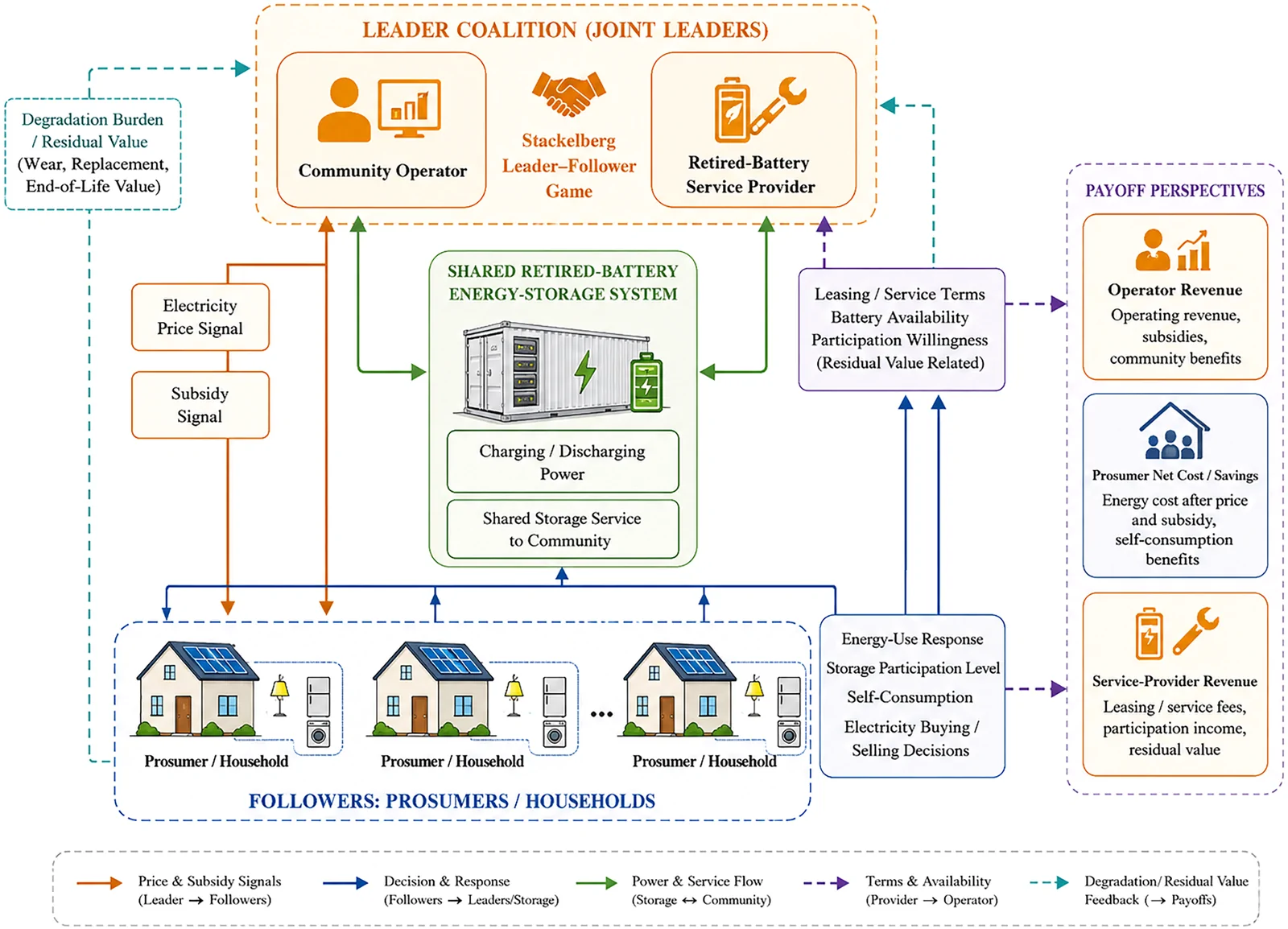
Strategy variables and game-theoretic relationships among the three community actors The community operator and retired-battery service provider act as leaders by determining pricing, subsidy, and storage-service decisions, while prosumers respond through energy-use and storage-participation strategies. Their interactions jointly determine the Stackelberg equilibrium and community welfare.

**Figure 4:**
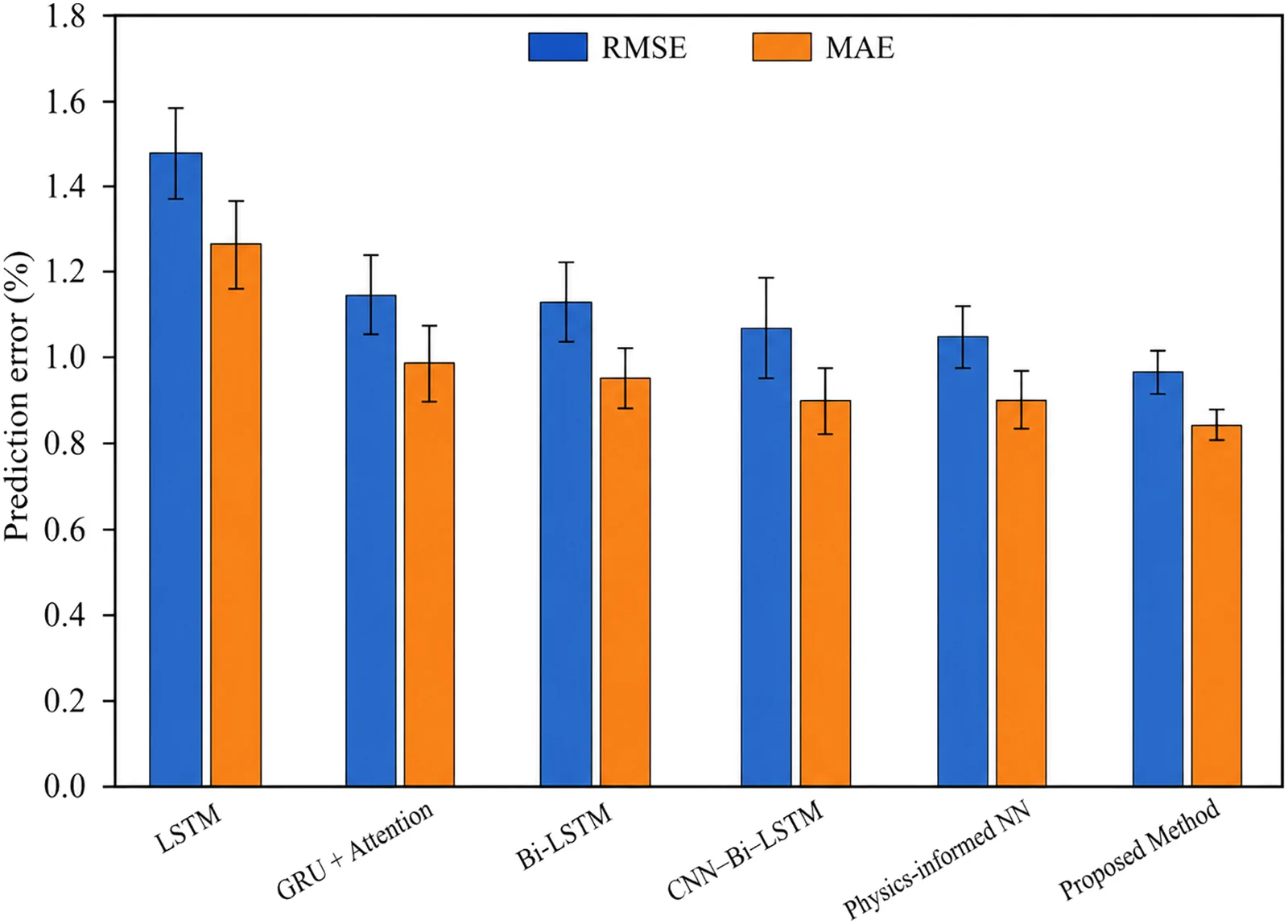
Comparison of capacity-degradation prediction performance among different methods The figure compares the prediction performance of different methods using RMSE, MAE, and R^2^. The proposed method achieves the best overall performance, indicating its stronger ability to capture nonlinear degradation patterns in heterogeneous retired batteries. Data are represented as mean ± SD from 10 repeated runs using random seeds 1–10.

The proposed model was configured using the physical-prior and training parameters summarized in Table 1, and the experimental dataset and community scenario settings are detailed in Table 2. The integrated framework linking capacity-degradation prediction, residual-value mapping, and game-theoretic subsidy allocation is illustrated in Figure 1; the prediction model architecture and the strategic relationships among the three community actors are shown in Figures 2 and 3, respectively. The prediction accuracy was evaluated against LSTM, GRU + Attention, Bi-LSTM, CNN-Bi-LSTM, and a physics-informed neural network (Figure 4 and Table 3). The proposed method achieves a root-mean-square error (RMSE) of 0.97% ± 0.04%, a mean absolute error (MAE) of 0.84% ± 0.03%, and R^2^ of 0.960 ± 0.002, outperforming all baselines. Relative to the strongest baseline (physics-informed NN and RMSE of 1.05%), the proposed model reduces RMSE by 7.6% and improves R^2^ from 0.948 to 0.960.

**Table 1:** Key physical-prior and training parameters of the capacity-degradation prediction model

Parameter	Meaning	Value range
*Α*	cycling-aging coefficient	0.0008–0.0015
*Β*	cycling-aging exponent	0.50–0.75
*Γ*	calendar-aging coefficient	0.0002–0.0006
*E*_*a*_	activation energy (kJ/mol)	24–32
*Θ*	current-stress exponent	1.10–1.45
*λ*_1_	physics-constraint weight	0.10–0.30
*λ*_2_	monotonicity regularization weight	0.05–0.15
*λ*_3_	cross-chemistry alignment weight	0.05–0.15
*Q*_*t*h_	end-of-life threshold	0.5·*Q*_0_

**Table 2:** Key settings of the experimental dataset and community scenario

Category	Item	Value	Source/basis
Battery	total battery aging samples	240	mixed battery-aging dataset (170 author-measured retired battery modules and 70 samples from public datasets^36^^,^^37^)
Battery	SOH range	50%–82%	operating envelope of the adopted retired-battery dataset
Battery	chemistry	LFP, NMC, and NCA	chemistry types covered by the mixed dataset
Battery	cycling temperature	20°C–35°C	temperature range observed in the adopted dataset
Battery	discharge rate	0.25C–1C	operating range observed in the adopted battery-aging dataset
Community	number of prosumers	60	typical small residential community scenario/community operating records
Community	total PV capacity	245 kWp	community operating records/scenario configuration
Community	storage capacity	380 kWh (cascade)	retired-battery storage configuration for the community scenario
Community	dispatch time slots	96 slots/day	15-min dispatch interval over 24 h
Community	time-of-use price	0.32/0.65/1.05 CNY/kWh	local time-of-use tariff/community operating records
Computation	solver	Gurobi 11.0	mixed-integer optimization solver used in the simulation

**Table 3:** Comparison of capacity-degradation prediction accuracy

Method	RMSE/%	MAE/%	R^2^
LSTM	1.48 ± 0.09	1.26 ± 0.08	0.918 ± 0.004
GRU (gated recurrent unit) + Attention	1.14 ± 0.07	0.98 ± 0.07	0.934 ± 0.002
Bi-LSTM (bidirectional LSTM)	1.13 ± 0.07	0.95 ± 0.05	0.941 ± 0.004
CNN-Bi-LSTM (convolutional neural network)	1.06 ± 0.08	0.90 ± 0.06	0.945 ± 0.003
Physics-informed NN (neural network)	1.05 ± 0.05	0.90 ± 0.05	0.948 ± 0.003
Proposed method	0.97 ± 0.04	0.84 ± 0.03	0.960 ± 0.002

**Figure 5:**
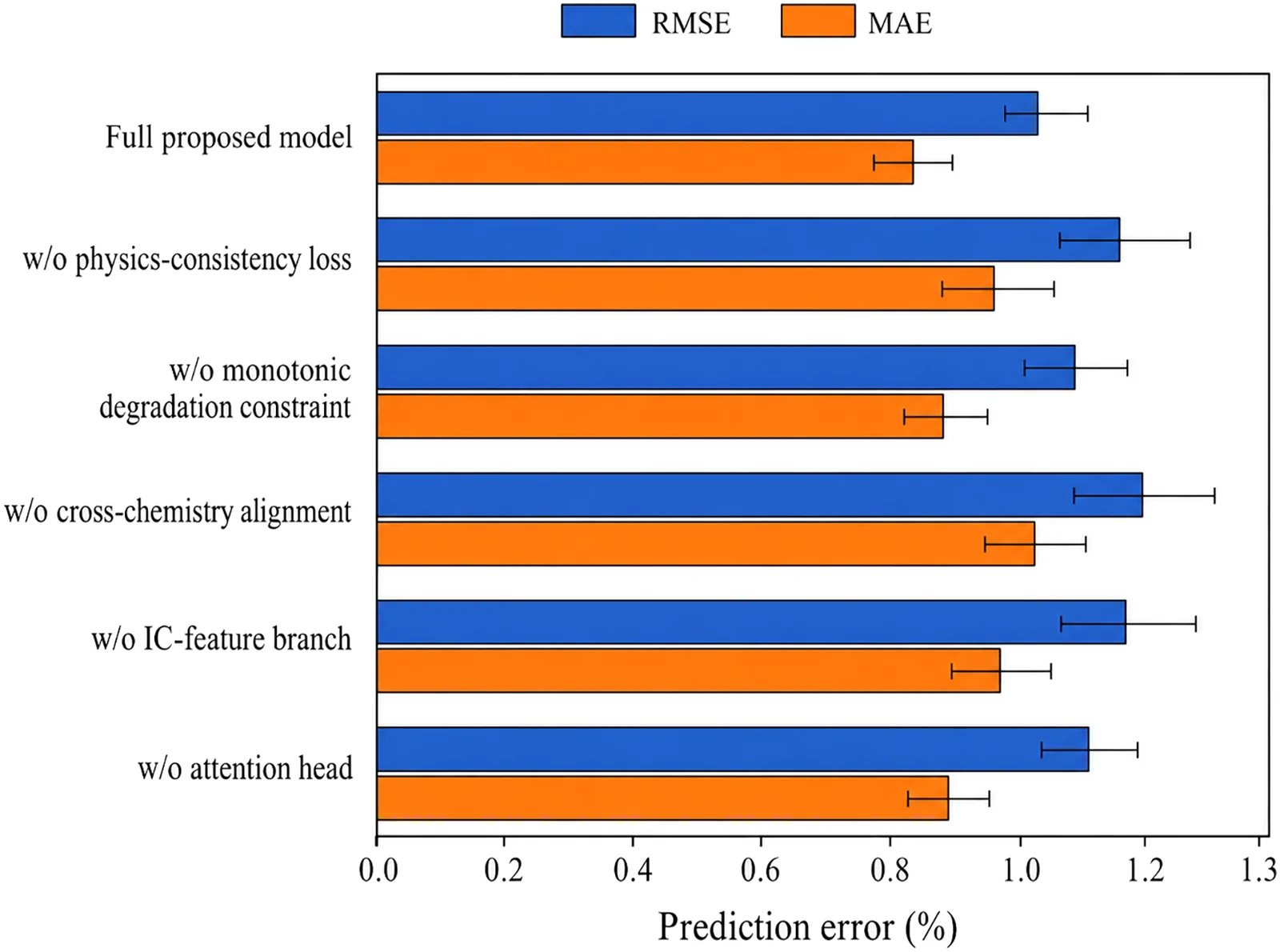
Ablation analysis of prediction errors for the proposed capacity-degradation model The figure compares the RMSE and MAE values of the full model and its ablated variants. The full model achieves the best performance, while removing key components leads to higher prediction errors, confirming the effectiveness of each module. Data are represented as mean ± SD from 10 repeated runs using random seeds 1–10.

Ablation analysis confirms the contribution of each component (Figure 5 and Table 4). Removing cross-chemistry alignment causes the largest degradation (RMSE of 1.12% and R^2^ of 0.940), followed by the IC-feature branch (RMSE 1.09%), physics-consistency loss (RMSE of 1.08%), attention head (RMSE of 1.04%), and monotonic constraint (RMSE of 1.03%).

**Table 4:** Ablation analysis of the proposed capacity-degradation prediction model

Variant	RMSE/%	MAE/%	R^2^
Full proposed model	0.97 ± 0.04	0.84 ± 0.03	0.960 ± 0.002
w/o physics-consistency loss	1.08 ± 0.06	0.92 ± 0.05	0.946 ± 0.003
w/o monotonic degradation constraint	1.03 ± 0.05	0.88 ± 0.04	0.953 ± 0.003
w/o cross-chemistry alignment	1.12 ± 0.06	0.96 ± 0.05	0.940 ± 0.004
w/o IC-feature branch	1.09 ± 0.07	0.94 ± 0.05	0.944 ± 0.004
w/o attention head	1.04 ± 0.05	0.89 ± 0.04	0.951 ± 0.003

**Figure 6:**
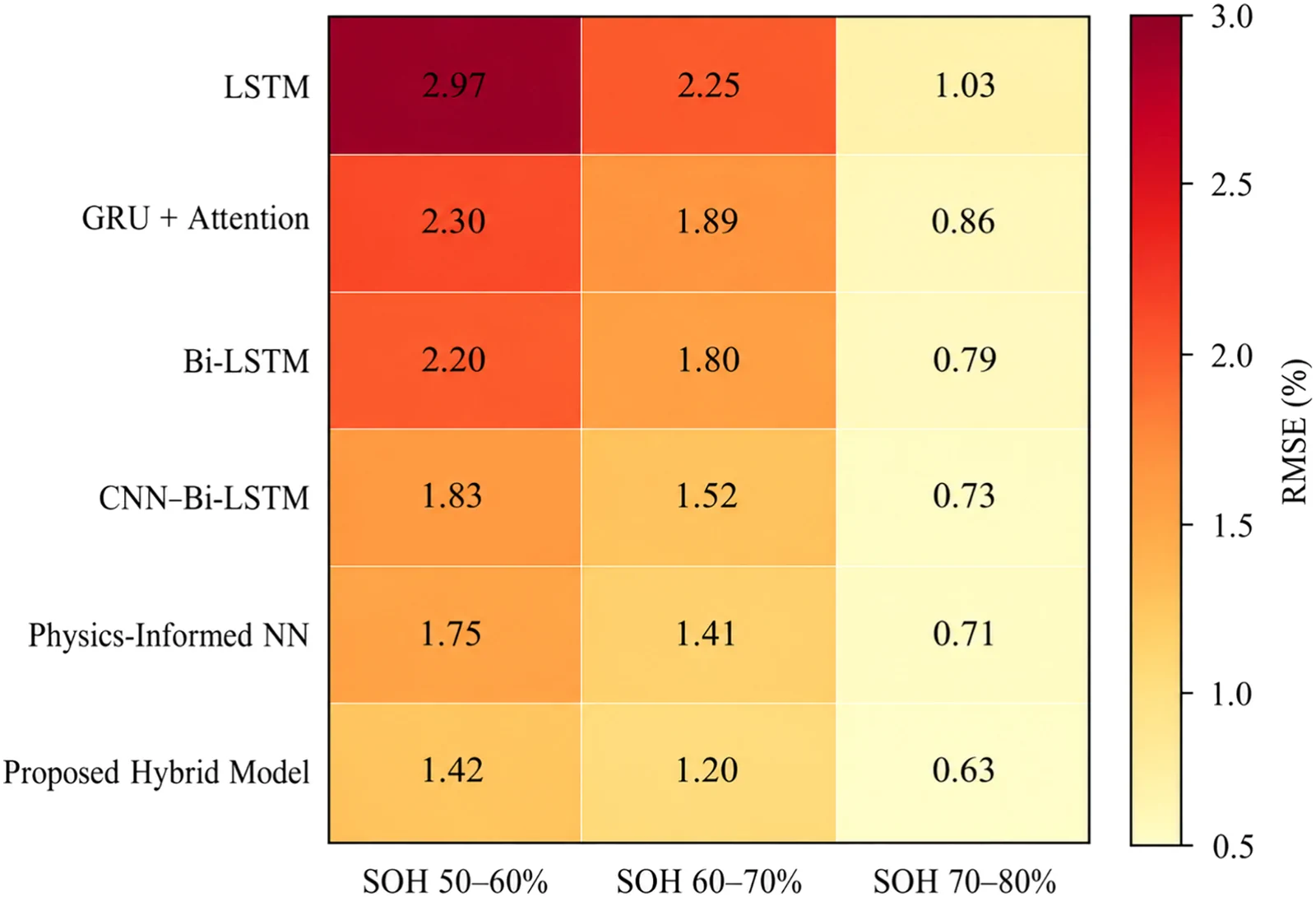
Heatmap of mean prediction RMSE across different SOH intervals The figure shows the distribution of mean prediction RMSE values for different methods across three SOH intervals. Prediction errors increase as SOH decreases, while the proposed hybrid model maintains the lowest RMSE across all battery-health conditions. Values represent mean RMSE across 10 repeated runs using random seeds 1–10; the corresponding variability is reported as SD in Table 5.

Under varying SOH (State of Health) intervals (50%–60%, 60%–70%, and 70%–80%), the proposed model maintains the lowest RMSE across all ranges (Figure 6 and Table 5). Errors increase at lower SOH, yet the proposed model achieves RMSE of 1.42% ± 0.07%, 1.20% ± 0.07%, and 0.63% ± 0.04% for SOH 50%–60%, 60%–70%, and 70%–80%, respectively, reducing the low-SOH error by 18.9% versus the physics-informed NN.

**Table 5:** Comparison of prediction RMSE across different SOH intervals

Model	SOH 50%–60%	SOH 60%–70%	SOH 70%–80%
LSTM	2.97 ± 0.13	2.25 ± 0.11	1.03 ± 0.07
GRU + Attention	2.30 ± 0.16	1.89 ± 0.11	0.86 ± 0.04
Bi-LSTM	2.20 ± 0.11	1.80 ± 0.15	0.79 ± 0.04
CNN-Bi-LSTM	1.83 ± 0.07	1.52 ± 0.08	0.73 ± 0.06
Physics-informed NN	1.75 ± 0.12	1.41 ± 0.08	0.71 ± 0.05
Proposed hybrid model	1.42 ± 0.07	1.20 ± 0.07	0.63 ± 0.04

**Figure 7:**
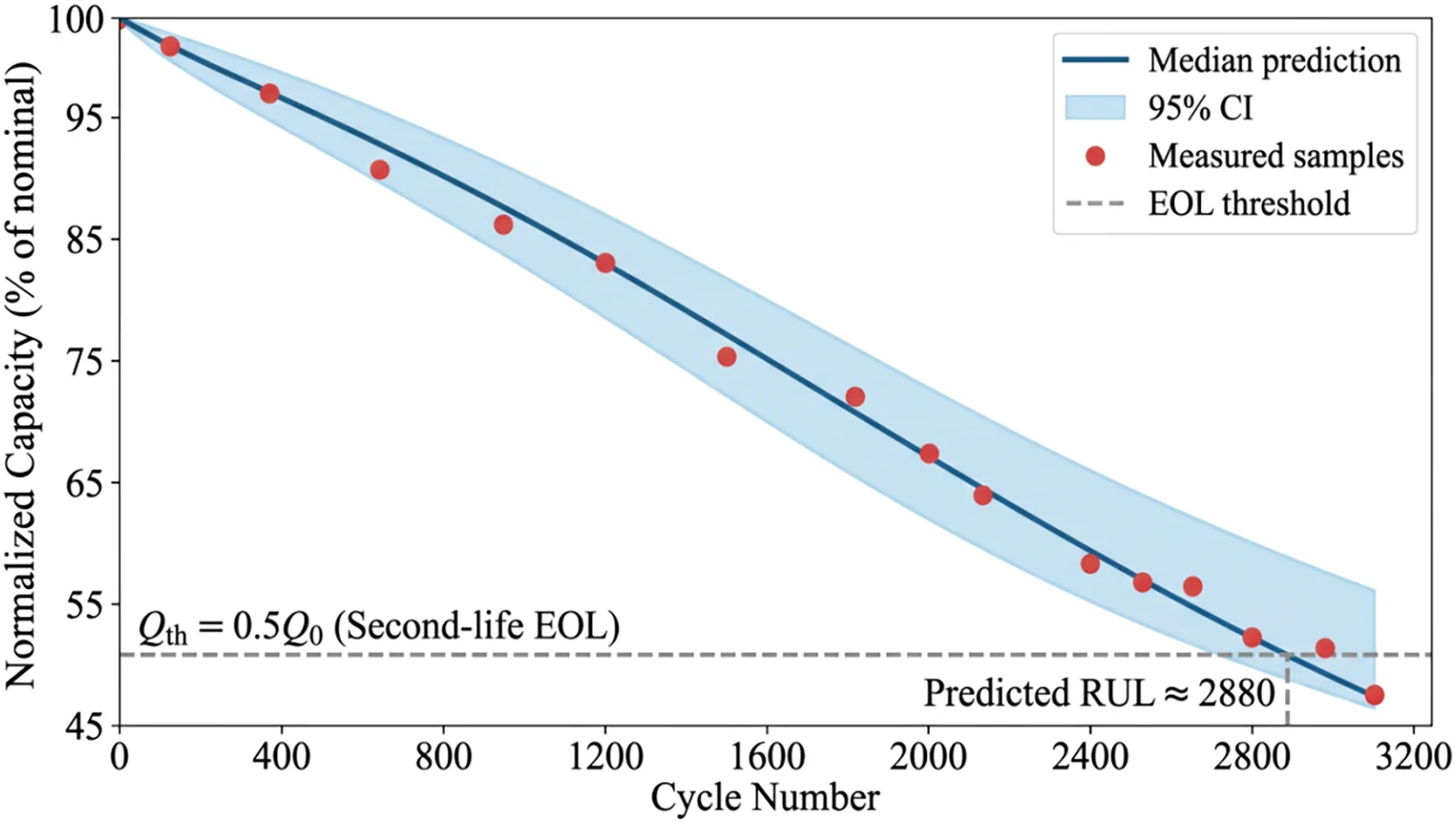
Comparison between predicted and measured capacity-degradation trajectories The figure compares the predicted capacity trajectory with the measured capacity samples over the full cycle range. The proposed model closely follows the observed degradation trend and provides the remaining useful life estimate through the second-life end-of-life threshold. The solid prediction curve represents the median, and the shaded area denotes the 95% confidence interval.

Long-term trajectory validation shows that the predicted median and 95% confidence interval closely track measured capacity samples (Figure 7). The predicted curve reaches the second-life EOL (end of life) threshold (Q_th_ = 0.5Q_0_) at approximately 2,880 cycles, providing the RUL basis for downstream residual-value mapping.

### Cross-chemistry transfer generalization

Cross-chemistry generalization is evaluated via an NMC (Nickel Manganese Cobalt) to-NCA (Nickel Cobalt Aluminum) transfer task (Table 6). Direct transfer without alignment yields RMSE of 1.54% ± 0.08% and R^2^ of 0.912 ± 0.005. Raw-feature MMD (maximum mean discrepancy) alignment reduces RMSE to 1.26% ± 0.06%. The proposed physics-constrained latent alignment further lowers RMSE to 1.05% ± 0.05% and raises R^2^ to 0.951 ± 0.003, outperforming raw-feature MMD by 16.7%. These results indicate that aligning degradation representations in the physics-constrained latent space better suppresses chemistry-specific noise than the raw-feature matching, supporting the proposed covariance-alignment term as an effective transfer regularization component.

**Table 6:** Representative cross-chemistry transfer prediction performance under the NMC-to-NCA task

Transfer setting	Method	RMSE/%	MAE/%	R^2^
NMC → NCA	direct transfer	1.54 ± 0.08	1.31 ± 0.07	0.912 ± 0.005
NMC → NCA	raw-feature MMD alignment	1.26 ± 0.06	1.07 ± 0.05	0.931 ± 0.004
NMC → NCA	proposed method	1.05 ± 0.05	0.89 ± 0.04	0.951 ± 0.003

### Dynamic residual-value evolution under different battery states

**Figure 8:**
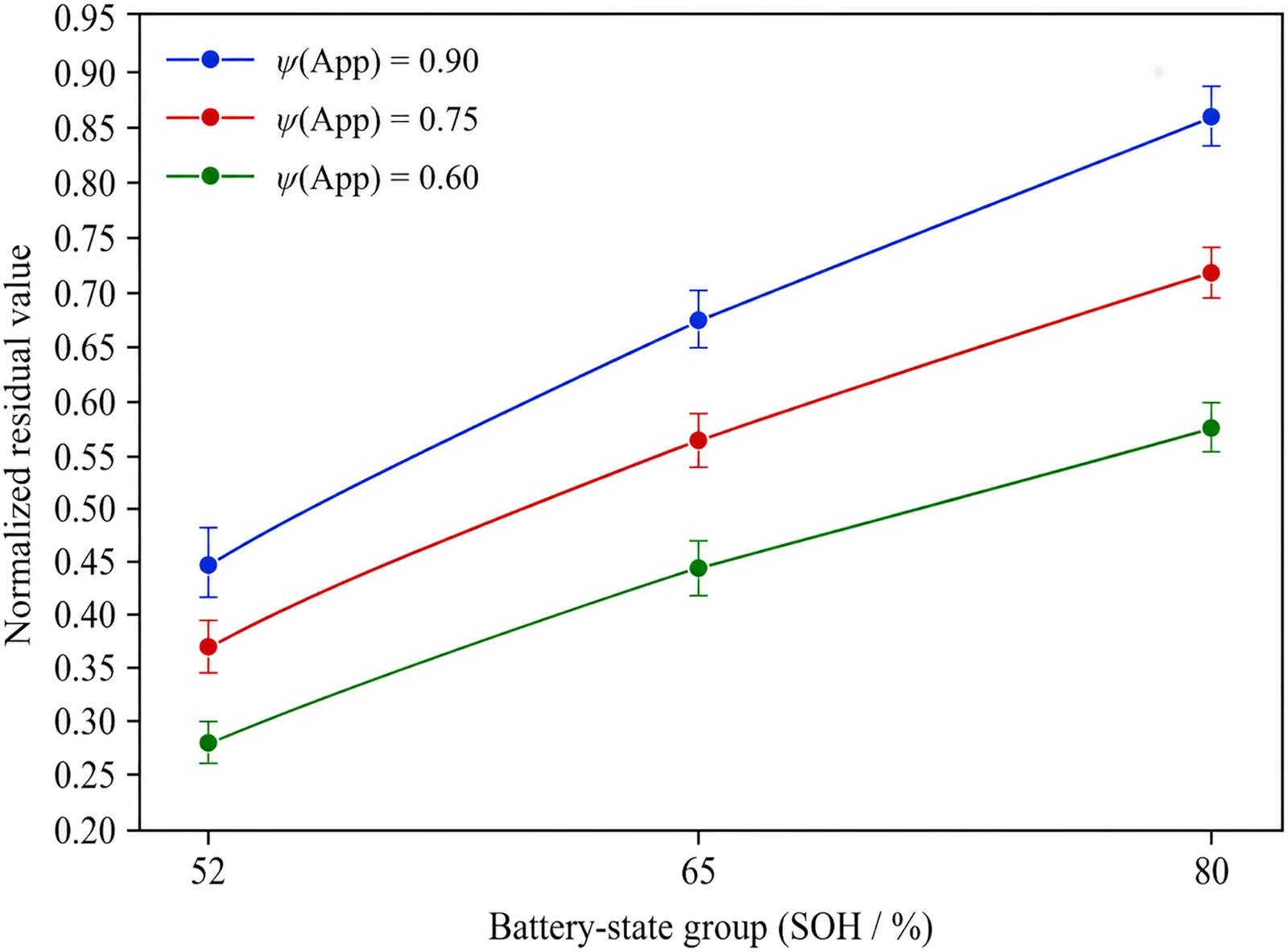
Normalized residual-value evolution under different SOH and scenario-adaptability levels The figure shows the normalized residual values of retired batteries under different SOH levels and scenario-adaptability coefficients. Residual value increases with both battery health state and application-scenario adaptability, indicating that the proposed mapping function can capture their joint effects. Data are represented as mean ± SD from 10 repeated runs using random seeds 1–10.

Residual-value mapping is evaluated across SOH levels of 52%, 65%, and 80% under scenario-adaptability coefficients ψ(App) = 0.60, 0.75, and 0.90 (Figure 8 and Table 7). With capacity and lifespan benchmarks normalized to 1 and predicted RUL held constant, the normalized residual value increases monotonically with both SOH and ψ(App). At ψ(App) = 0.90, residual value rises from 0.423 ± 0.025 (SOH 52%) to 0.864 ± 0.021 (SOH 80%); at fixed SOH 65%, it increases from 0.445 ± 0.020 to 0.667 ± 0.022 as ψ(App) increases from 0.60 to 0.90. These results confirm that the mapping function jointly captures battery-state differences and scenario adaptability, providing a quantitative residual-value signal for downstream payoff and subsidy-allocation modeling.

**Table 7:** Normalized residual-value mapping results under representative retired-battery states

Battery-state group (SOH/%)	Predicted RUL/cycles	Scenario adaptability *ψ*(App)	Normalized residual value
52	836 ± 87	0.9	0.423 ± 0.025
65	2,016 ± 96	0.9	0.667 ± 0.022
80	3,094 ± 126	0.9	0.864 ± 0.021
52	836 ± 87	0.75	0.353 ± 0.021
65	2,016 ± 96	0.75	0.556 ± 0.021
80	3,094 ± 126	0.75	0.720 ± 0.020
52	836 ± 87	0.6	0.282 ± 0.017
65	2,016 ± 96	0.6	0.445 ± 0.020
80	3,094 ± 126	0.6	0.576 ± 0.019

### Consistency between predicted degradation and residual-value mapping

**Figure 9:**
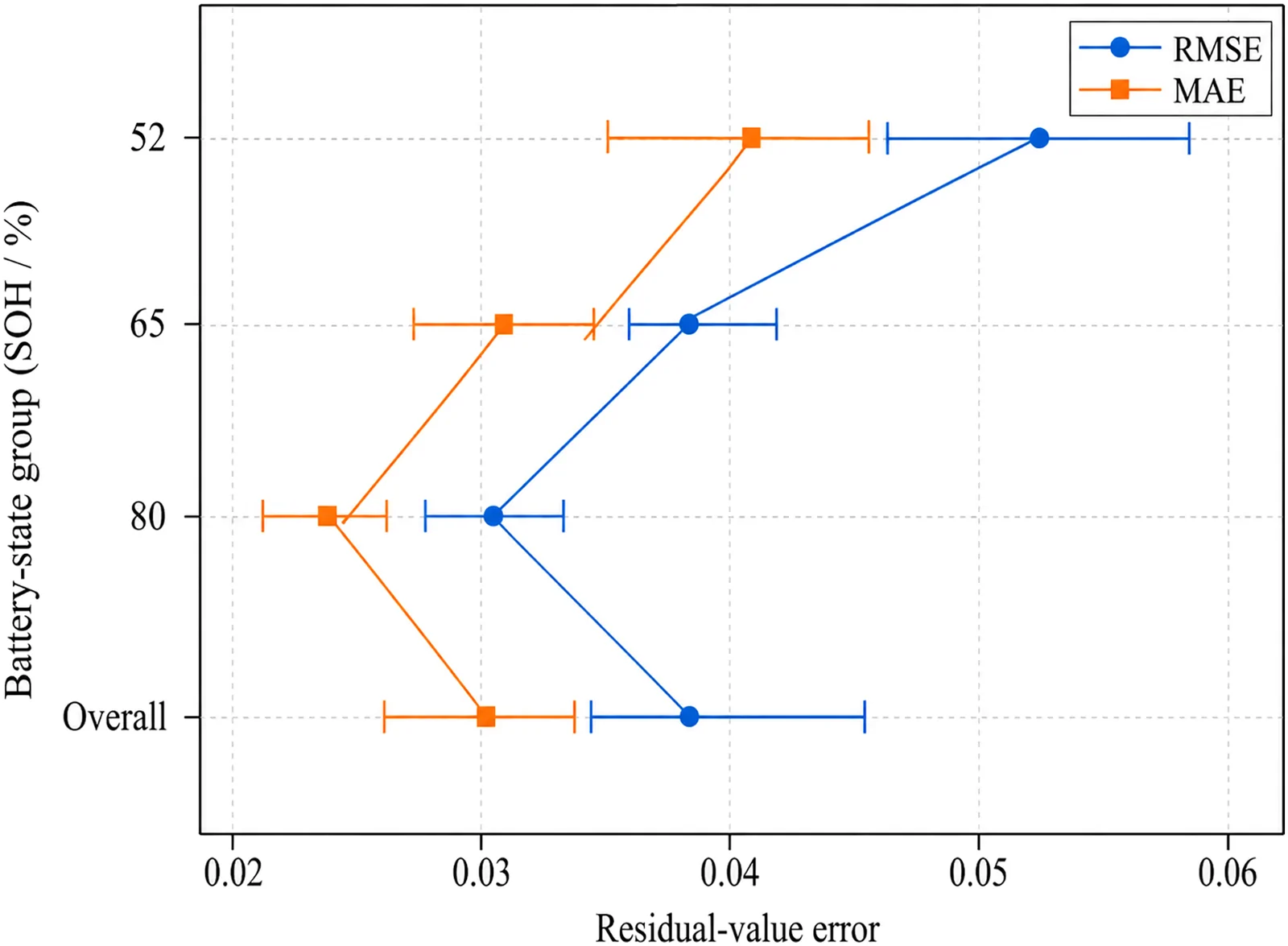
Residual-value consistency errors across different battery-state groups The figure compares the RMSE and MAE of residual values derived from predicted and measured degradation trajectories across different SOH groups. The consistently low errors indicate that the proposed mapping function reliably converts predicted battery degradation into residual-value signals. Data are represented as mean ± SD from 10 repeated runs using random seeds 1–10.

Residual-value mapping consistency is validated by comparing predicted and measured capacity trajectories across SOH levels of 52%, 65%, and 80% (Figure 9 and Table 8). Over 10 repeated trials, the overall RMSE and MAE between predicted and measured residual values are 0.041 ± 0.006 and 0.032 ± 0.005, respectively, with per-group RMSE decreasing from 0.052 ± 0.007 (SOH 52%) to 0.031 ± 0.004 (SOH 80%). These low errors confirm that the mapping function reliably translates predicted battery degradation into consistent economic signals, supporting its use in downstream payoff and subsidy-allocation calculations.

**Table 8:** Residual-value consistency between predicted and measured degradation trajectories

Battery-state group (SOH/%)	Residual-value RMSE	Residual-value MAE
52	0.052 ± 0.007	0.041 ± 0.006
65	0.038 ± 0.005	0.029 ± 0.004
80	0.031 ± 0.004	0.024 ± 0.003
Overall	0.041 ± 0.006	0.032 ± 0.005

### Degradation-burden characterization under alternative dispatch strategies

Degradation-burden sensitivity to dispatch intensity is evaluated through four representative strategies: conservative (0.28C, shallow cycling), balanced (0.46C, moderate DoD (Depth of Disch)), peak-shaving intensive (0.68C during peak hours), and aggressive (0.85C, deep cycling) (Table 9). The degradation burden index increases monotonically with dispatch intensity, from 0.31 ± 0.04 under conservative dispatch to 0.93 ± 0.08 under aggressive dispatch, with corresponding daily-equivalent capacity loss rising from 0.42‰ ± 0.05‰/day to 1.24‰ ± 0.10‰/day and residual-value reduction increasing from 0.18% ± 0.02% to 0.61% ± 0.05%. These results confirm that the degradation-burden index effectively captures health-cost variation across operational strategies, validating its incorporation into the game-theoretic payoff function.

**Table 9:** Battery-degradation burden under alternative dispatch strategies

Dispatch strategy	Average C-rate	Daily equivalent capacity loss (‰/day)	Degradation burden index	Residual-value reduction/%
Conservative	0.28C	0.42 ± 0.05	0.31 ± 0.04	0.18 ± 0.02
Balanced	0.46C	0.67 ± 0.06	0.50 ± 0.05	0.29 ± 0.03
Peak-shaving intensive	0.68C	0.96 ± 0.08	0.72 ± 0.07	0.43 ± 0.04
Aggressive	0.85C	1.24 ± 0.10	0.93 ± 0.08	0.61 ± 0.05

### Game equilibrium solution and community welfare allocation results

The Stackelberg equilibrium under the proposed dynamic allocation achieves a selling price of 0.84 CNY/kWh and a subsidy signal of 0.21 CNY/kWh, yielding operator revenue of 1,853 CNY/day, prosumer savings of 743 CNY/day (from 30.00 to 17.62 CNY/household), service-provider leasing revenue of 962 CNY/day at a utilization rate of 0.68, a self-consumption ratio of 71.4%, and a system-level carbon-reduction benefit of 52 CNY/day, with total community welfare reaching 3,610 CNY/day (Table 10).

**Table 10:** Key variables of the game equilibrium

Actor	Variable	Equilibrium value
Operator	daily average selling price	0.84 CNY/kWh
Operator	daily average subsidy signal	0.21 CNY/kWh
Operator	daily operating revenue	1,853 CNY/day
Prosumer	average self-consumption ratio	71.4%
Prosumer	baseline daily energy cost (no-subsidy benchmark)	30.00 CNY/household
Prosumer	average daily energy cost (this study)	17.62 CNY/household
Prosumer	total daily savings of 60 households	743 CNY/day
Service provider	average utilization *ρ*_*t*_	0.68
Service provider	daily leasing revenue	962 CNY/day
System	daily carbon-reduction benefit	52 CNY/day
System	total community welfare	3,610 CNY/day

**Figure 10:**
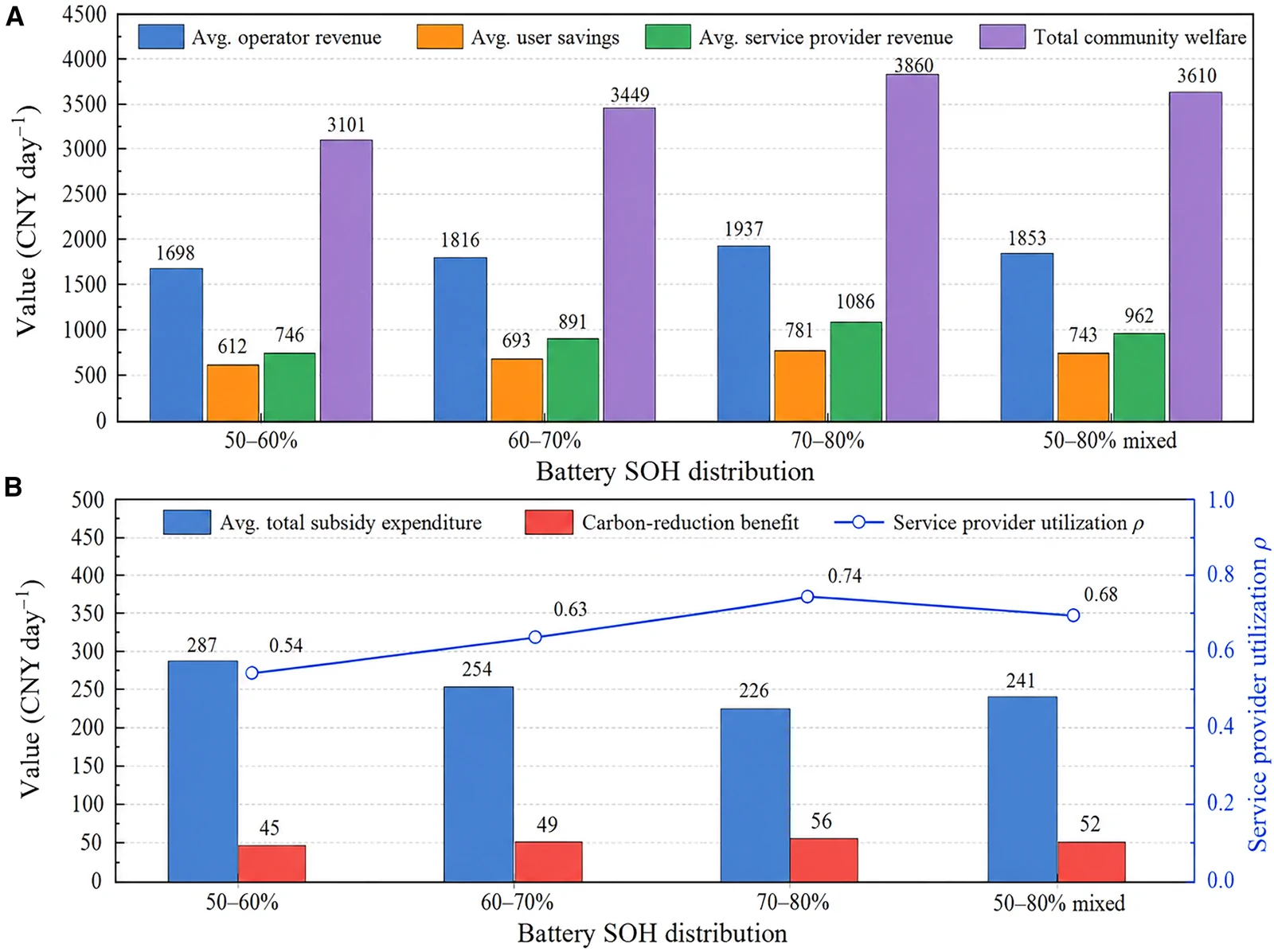
Equilibrium welfare and utilization outcomes under different retired-battery SOH distributions (A) Operator revenue, prosumer savings, service-provider revenue, and total community welfare. (B) Service-provider utilization and subsidy expenditure. Higher battery SOH improves utilization and welfare while reducing the subsidy required to support community operation. The figure compares operator revenue, prosumer savings, service-provider revenue, utilization, subsidy expenditure, and total community welfare under different SOH distributions. Higher battery SOH improves utilization and welfare while reducing the subsidy required to support community operation.

Under varying SOH distributions, equilibrium outcomes improve monotonically with battery health (Figure 10 and Table 11). Total welfare increases from 3,101 CNY/day (SOH 50%–60%) to 3,860 CNY/day (SOH 70%–80%), while required subsidy expenditure decreases from 287 to 226 CNY/day; the mixed 50%–80% scenario yields 3,610 CNY/day at a utilization rate of 0.68. These results confirm that SOH-dependent residual-value signals are necessary for accurate game-theoretic allocation.

**Table 11:** Equilibrium simulation results under different retired-battery SOH distributions

Battery SOH distribution	Avg. operator revenue/CNY day^−1^	Avg. total subsidy expenditure/CNY day^−1^	Avg. user savings/CNY day^−1^	Avg. service provider revenue/CNY day^−1^	Service provider utilization ρ	Carbon-reduction benefit/CNY day^−1^	Total community welfare/CNY day^−1^
50%–60%	1,698	287	612	746	0.54	45	3,101
60%–70%	1,816	254	693	891	0.63	49	3,449
70%–80%	1,937	226	781	1,086	0.74	56	3,860
50%–80% mixed	1,853	241	743	962	0.68	52	3,610

**Figure 11:**
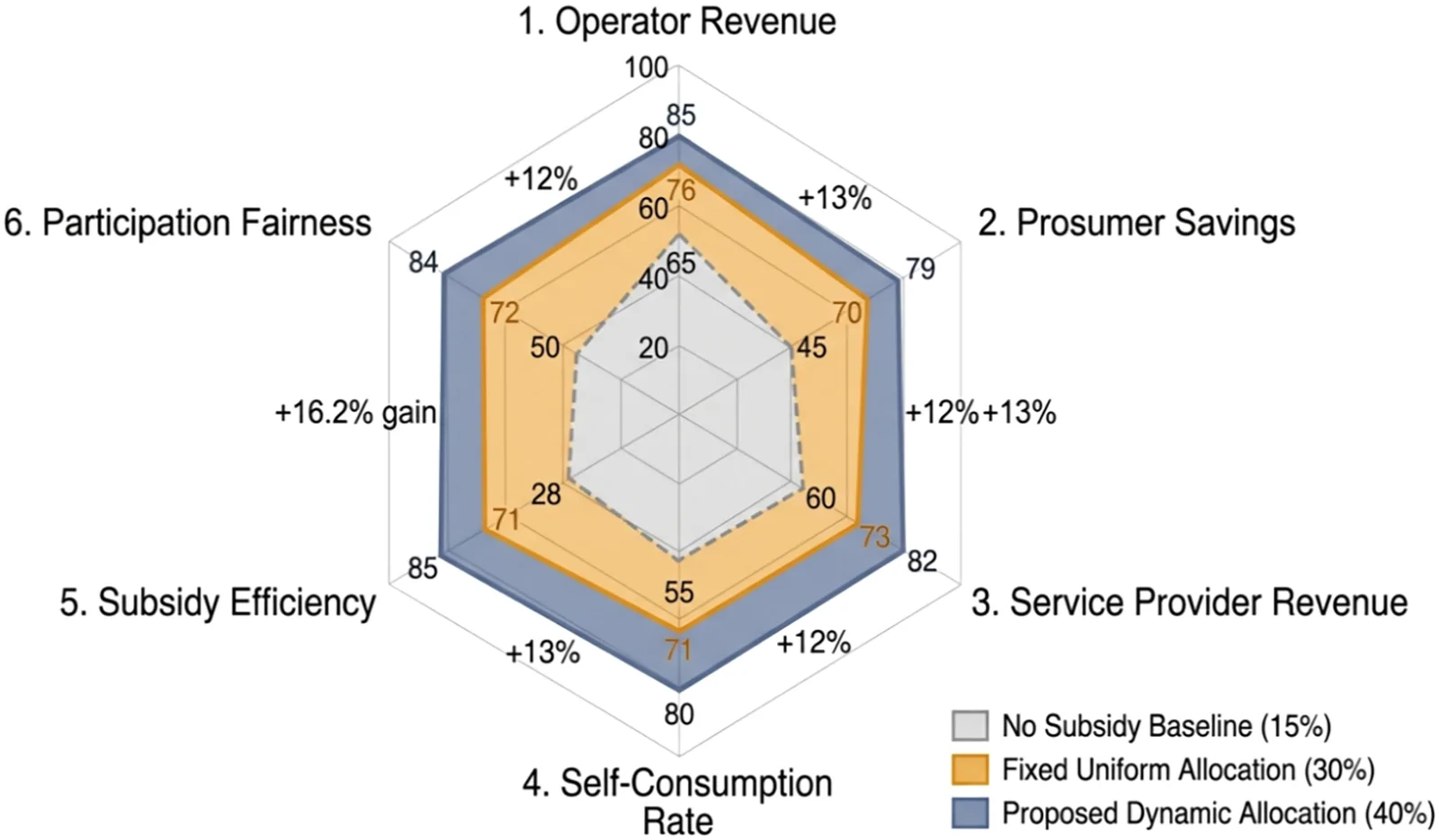
Six-dimensional comparison of welfare-allocation performance The radar chart compares the no-subsidy baseline, fixed uniform allocation, and proposed dynamic allocation across six economic and operational indicators. The proposed mechanism achieves the best overall performance, particularly in subsidy efficiency and participation fairness.

A six-dimensional comparison of the no-subsidy baseline, fixed uniform allocation, and proposed dynamic scheme across operator revenue, prosumer savings, service-provider revenue, self-consumption rate, subsidy efficiency, and participation fairness shows the proposed mechanism dominating all dimensions (Figure 11). Relative to fixed uniform allocation, the dynamic scheme improves subsidy efficiency from 71 to 85 and participation fairness from 72 to 84, indicating that redistribution by degradation contribution, participation duration, and circular-economy correction factors achieves superior coordination of economic benefits and incentive fairness.

### Sensitivity to residual-value mapping weights

**Figure 12:**
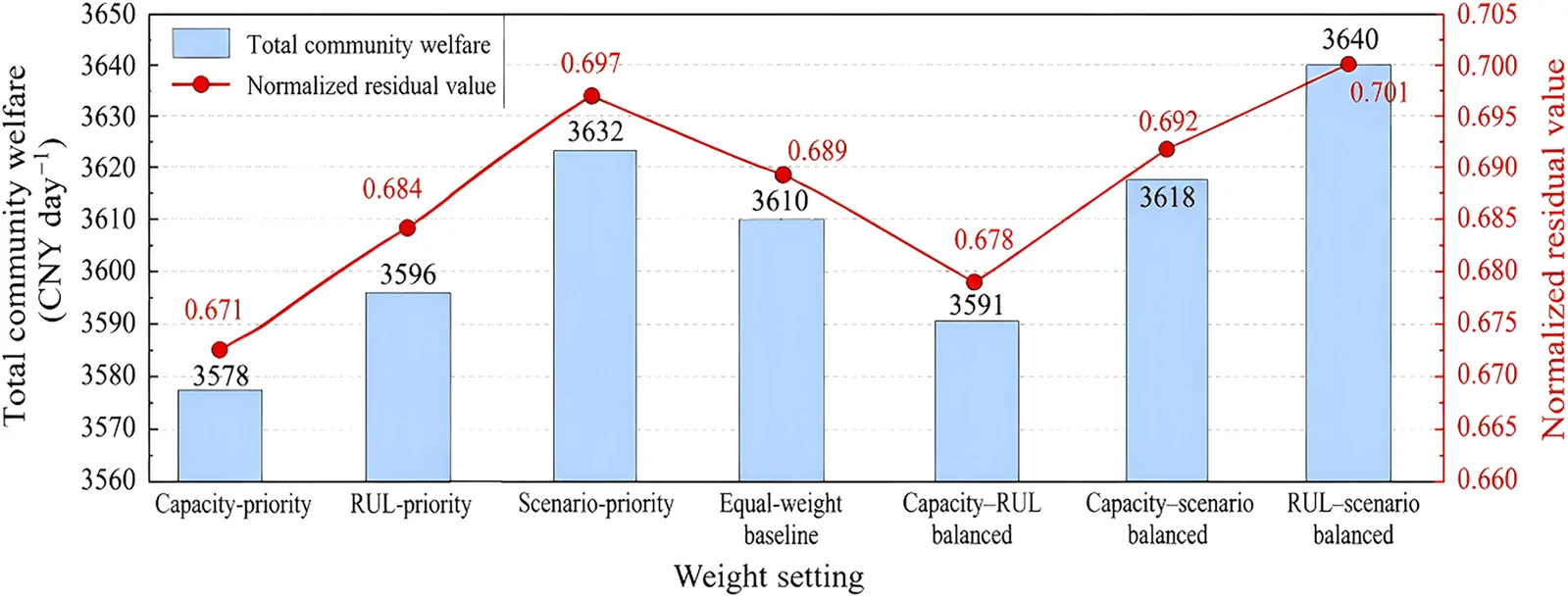
Sensitivity of residual-value mapping outcomes to different weight settings The figure compares normalized residual value and total community welfare under alternative weighting schemes for capacity, RUL, and scenario adaptability. The limited variation across settings indicates that the proposed mechanism is relatively robust to changes in the residual-value weights.

Sensitivity to residual-value mapping weights is examined by varying ω_1_ (capacity), ω_2_ (RUL), and ω_3_ (scenario adaptability) across seven settings against an equal-weight baseline (Figure 12 and Table 12). Total community welfare remains stable within 3,578–3,640 CNY/day, with a maximum deviation of only 62 CNY/day (1.7%). The RUL-scenario-balanced setting achieves the highest welfare at 3,640 CNY/day, a normalized residual value of 0.701, and a utilization rate of 0.70, whereas the capacity-priority setting yields the lowest welfare at 3,578 CNY/day. These results indicate that RUL and scenario adaptability exert stronger downstream effects on community operation than current capacity alone and that residual-value mapping weights primarily influence valuation preference without compromising the feasibility or stability of the game equilibrium.

**Table 12:** Sensitivity of residual-value mapping results to weight settings

Weight setting	*ω*_1_: Capacity	*ω*_2_: RUL	*ω*_3_: Scenario adaptability	Normalized residual value	Service-provider utilization *ρ*	Total community welfare/CNY day^−1^
Capacity priority	0.5	0.3	0.2	0.671	0.66	3,578
RUL priority	0.3	0.5	0.2	0.684	0.67	3,596
Scenario priority	0.3	0.2	0.5	0.697	0.69	3,632
Equal-weight baseline	0.33	0.33	0.33	0.689	0.68	3,610
Capacity-RUL balanced	0.4	0.4	0.2	0.678	0.67	3,591
Capacity-scenario balanced	0.4	0.2	0.4	0.692	0.68	3,618
RUL-scenario balanced	0.2	0.4	0.4	0.701	0.7	3,640

### Sensitivity to circular-economy correction weights

**Figure 13:**
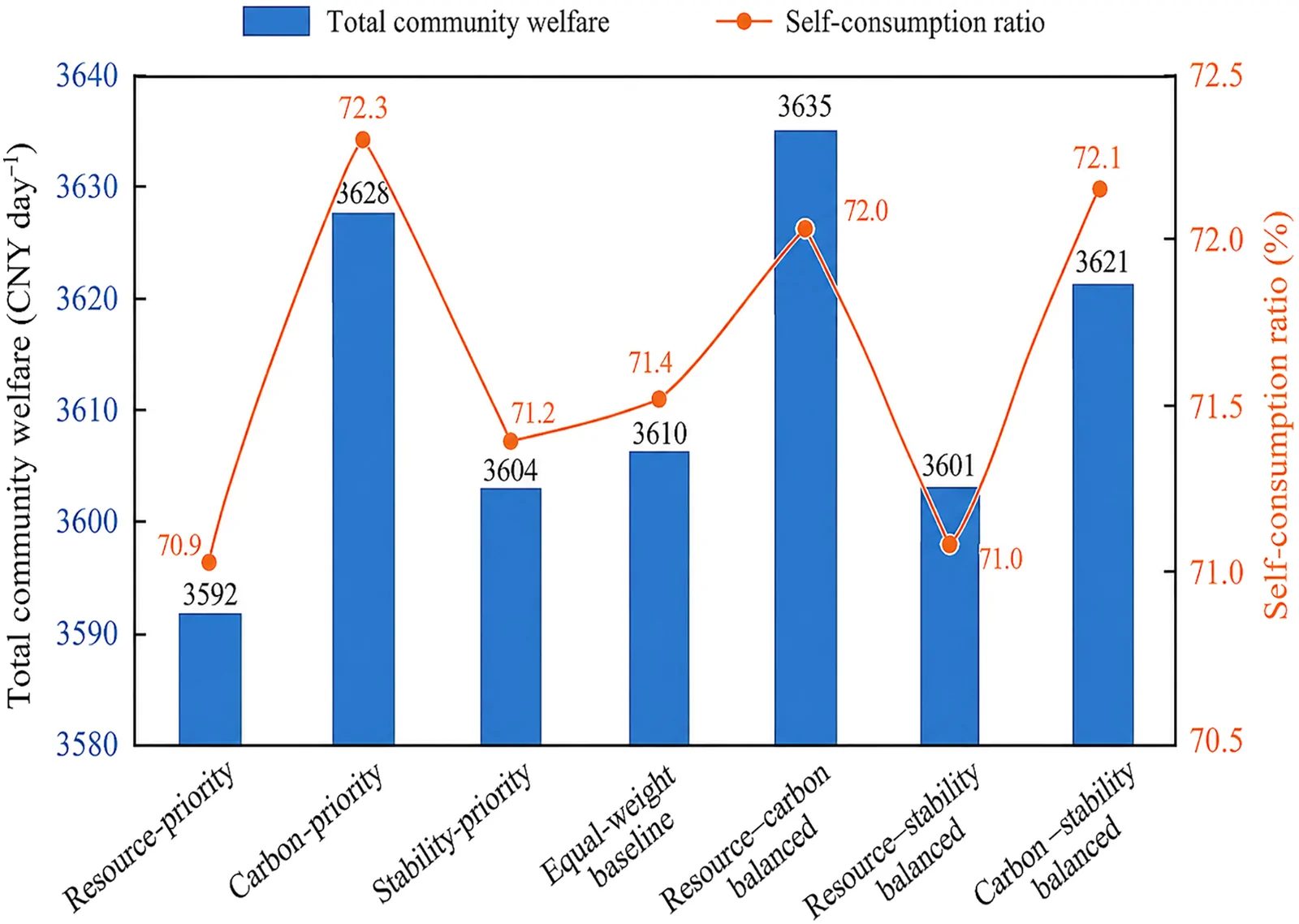
Sensitivity of allocation performance to circular-economy correction weights The figure compares total community welfare and self-consumption ratio under different circular-economy correction-weight settings. The limited variation across scenarios indicates that the proposed allocation mechanism remains robust to changes in policy-oriented priorities.

Sensitivity to circular-economy correction weights is examined by varying η_1_ (resource recovery), η_2_ (carbon reduction), and η_3_ (community stability) across seven scenarios against an equal-weight baseline (Figure 13 and Table 13). Total community welfare remains stable within 3,592–3,635 CNY/day, with the resource-carbon-balanced setting achieving the highest welfare at 3,635 CNY/day and the carbon-priority setting yielding the highest self-consumption ratio at 72.3%. Average subsidy expenditure stays within a narrow band of 238–246 CNY/day, indicating that weight adjustment does not induce excessive subsidy fluctuation. These results confirm that circular-economy correction weights primarily influence allocation preference rather than the feasibility or economic stability of the equilibrium solution.

**Table 13:** Sensitivity of allocation performance to circular-economy correction weights

Weight setting	*η*_1_	*η*_2_	*η*_3_	Total community welfare/CNY day^−1^	Self-consumption ratio/%	Avg. total subsidy expenditure/CNY day^−1^
Resource priority	0.5	0.25	0.25	3,592	70.9	238
Carbon priority	0.25	0.5	0.25	3,628	72.3	244
Stability priority	0.25	0.25	0.5	3,604	71.2	246
Equal-weight baseline	0.33	0.33	0.33	3,610	71.4	241
Resource-carbon balanced	0.4	0.4	0.2	3,635	72.0	242
Resource-stability balanced	0.4	0.2	0.4	3,601	71.0	240
Carbon-stability balanced	0.2	0.4	0.4	3,621	72.1	245

### Sensitivity to the degradation threshold and penalty coefficient

Sensitivity to the degradation threshold ΔQ_th_ and penalty coefficient ηd is examined through one-factor-at-a-time analysis, with ΔQ_th_ varied from 0.60‰/day to 1.00‰/day and η_d_ = 2.0 from 1.0 to 3.0 around the baseline (ΔQ_th_ = 0.80‰/day, η_d_ = 2.0) (Table 14).

**Table 14:** Sensitivity of subsidy expenditure, degradation burden, and community welfare to Δ*Q*^*th*^ and *η*_*d*_

Parameter setting	Δ*Q*^*th*^ (‰/day)	*η*_*d*_	Total subsidy expenditure (CNY/day)	Degradation burden index	Total community welfare (CNY/day)
Strict threshold	0.60	2.0	236	0.44	3,568
Weak penalty	0.80	1.0	245	0.56	3,594
Baseline	0.80	2.0	241	0.50	3,610
Strong penalty	0.80	3.0	238	0.46	3,581
Lenient threshold	1.00	2.0	246	0.57	3,589

Stricter thresholds reduce both the degradation burden index (from 0.50 to 0.44) and subsidy expenditure (from 241 to 236 CNY/day) yet prematurely restrict economically beneficial storage participation, lowering total welfare to 3,568 CNY/day. Conversely, a lenient threshold (1.00‰/day) raises the burden index to 0.57 and subsidy expenditure to 246 CNY/day without improving welfare (3,589 CNY/day). Weakening the penalty (η_d_ = 1.0) increases the burden index to 0.56 and subsidy expenditure to 245 CNY/day, whereas strengthening it (η_d_ = 3.0) suppresses the burden to 0.46 and subsidies to 238 CNY/day but also dampens participation incentives, reducing welfare to 3,581 CNY/day.

The baseline combination achieves the highest welfare at 3,610 CNY/day. Total welfare remains within 3,568–3,610 CNY/day, with a maximum deviation of approximately 1.2%, indicating that community welfare is relatively stable under moderate parameter variations and that the baseline setting provides a balanced compromise among battery-lifetime protection, subsidy expenditure, and storage-participation incentives.

### Sensitivity to subsidy intensity

Sensitivity to subsidy intensity is evaluated across five levels (0.10–0.40 CNY/kWh) comparing the no-subsidy baseline, fixed uniform allocation, and proposed dynamic mechanism (Table 15). The no-subsidy baseline remains constant at 2,822 CNY/day. Fixed uniform allocation peaks at 3,137 CNY/day under ζ = 0.15 CNY/kWh but declines to 2,577 CNY/day at ζ = 0.40, indicating that excessive uniform subsidies induce overcompensation and efficiency loss. The proposed dynamic mechanism consistently outperforms both alternatives across all intensities, rising from 3,198 CNY/day at ζ = 0.10 to a maximum of 3,610 CNY/day at ζ = 0.21 and then decreasing to 3,364 CNY/day at ζ = 0.40. These results demonstrate that moderate subsidy intensity maximizes welfare and that the dynamic allocation mechanism, by redistributing subsidies according to degradation contribution, participation duration, and circular-economy correction factors, exhibits superior robustness to subsidy-intensity variation compared with uniform distribution.

**Table 15:** Comparison of community welfare allocation under different subsidy intensities (CNY/day)

Subsidy intensity *ζ* (CNY/kWh)	No subsidy	Fixed uniform	This study
0.10	2,822	3,005	3,198
0.15	2,822	3,137	3,409
0.21	2,822	3,054	3,610
0.30	2,822	2,891	3,531
0.40	2,822	2,577	3,364

### Robustness under perturbation scenarios

**Figure 14:**
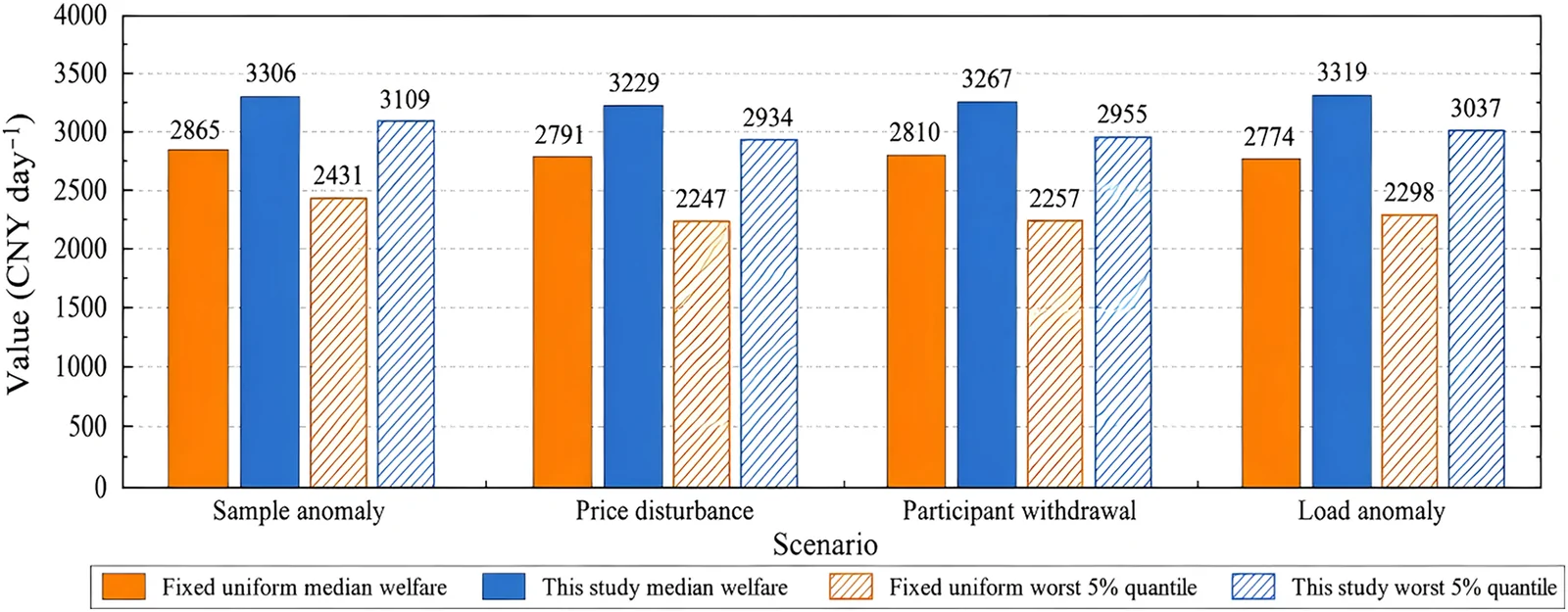
Robustness comparison of welfare performance under multiple perturbation scenarios The figure compares the median welfare and worst 5% welfare quantile of the fixed uniform scheme and proposed dynamic allocation method under multiple perturbation scenarios. The proposed method consistently achieves higher welfare, demonstrating stronger operational robustness. The median denotes the 50th percentile, and the worst 5% welfare quantile denotes the 5th percentile of welfare outcomes under each perturbation scenario.

Robustness is evaluated under four perturbation scenarios—sample anomaly, price disturbance, participant withdrawal, and load anomaly—using fixed uniform allocation as the baseline (Figure 14 and Table 16). The proposed dynamic mechanism consistently achieves higher median welfare across all scenarios: 3,306 vs. 2,865 CNY/day under sample anomaly, 3,229 vs. 2,791 CNY/day underprice disturbance, 3,267 vs. 2,810 CNY/day under participant withdrawal, and 3,319 vs. 2,774 CNY/day under load anomaly. Worst-case protection is also superior, with the 5% quantile rising from 2,431 to 3,109, 2,247 to 2,934, 2,257 to 2,955, and 2,298 to 3,037 CNY/day, respectively. Interquartile range is reduced in all cases (e.g., 523 to 308 under price disturbance), indicating smaller welfare fluctuation. Solving time remains approximately 15 s across all scenarios, confirming that the additional computational cost is acceptable for day-ahead or rolling community scheduling. These results demonstrate that the proposed mechanism improves both welfare robustness and downside-risk protection under multiple operational uncertainties.

**Table 16:** Comparison of robustness indicators across multiple perturbation scenarios

Scenario	Scheme	Median welfare/CNY day^−1^	IQR/CNY day^−1^	Worst 5% quantile/CNY day^−1^	Solving time/s
Sample anomaly	fixed uniform	2,865	412	2,431	13.8
Sample anomaly	this study	3,306	268	3,109	15.1
Price disturbance	fixed uniform	2,791	523	2,247	13.6
Price disturbance	this study	3,229	308	2,934	15.3
Participant withdrawal	fixed uniform	2,810	521	2,257	13.4
Participant withdrawal	this study	3,267	314	2,955	15
Load anomaly	fixed uniform	2,774	463	2,298	13.9
Load anomaly	this study	3,319	281	3,037	15.2

### Robustness to alternative decision-making sequences

Robustness to alternative decision-making sequences is examined by comparing five structures with identical payoff functions, residual-value mapping, and operational constraints (Table 17). The proposed operator-service-provider leader coalition achieves the highest total community welfare at 3,610 CNY/day, outperforming operator-led (3,556), service-provider-led (3,518), prosumer-led reverse (3,472), and simultaneous-move (3,424) structures. While revenue distribution shifts across sequences—service-provider-led elevates provider revenue to 1,004 CNY/day at the expense of system coordination, and prosumer-led raises user savings to 777 CNY/day while depressing operator and provider revenues—the proposed coalition maintains the best overall balance. These results confirm that the selected Stackelberg hierarchy is practically reasonable and that incorporating residual-value signals and degradation-burden terms into upper level incentive design remains beneficial regardless of decision-sequence variation.

**Table 17:** Comparison of equilibrium outcomes under alternative decision-making sequences

Decision structure	First mover(s)	Response actor(s)	Operator revenue/CNY day^−1^	User savings/CNY day^−1^	Service-provider revenue/CNY day^−1^	Total community welfare/CNY day^−1^
Proposed leader coalition	operator + service provider	prosumers	1,853	743	962	3,610
Operator-led Stackelberg	operator	service provider + prosumers	1,847	721	936	3,556
Service-provider-led Stackelberg	service provider	operator + prosumers	1,796	697	1,004	3,518
Prosumer-led reverse Stackelberg	prosumers	operator + service provider	1,728	777	865	3,472
Simultaneous-move benchmark	all actors simultaneously	–	1,762	708	902	3,424

## Discussion

The proposed framework addresses a structural disconnect in the second-life battery literature: capacity-degradation predictions have traditionally remained as isolated end indicators, while community-level incentive designs have treated battery health as an exogenous constant. By embedding a physics-constrained transfer-learning predictor within a dynamic residual-value mapping and Stackelberg subsidy game, this study demonstrates that prediction accuracy can be propagated directly into economic coordination. The hybrid model achieves sub-1% RMSE and strong cross-chemistry generalization, yet the more consequential finding is that these physical-state estimates can be stably translated into economically interpretable value signals. Ablation and consistency analyses confirm that neither pure data-driven nor purely physics-based approaches alone are sufficient for heterogeneous retired batteries; rather, the integration of electrochemical priors, cross-domain alignment, and attention-based temporal modeling is necessary to bridge the gap between laboratory cycling data and field residual-value assessment.

The residual-value mapping experiments reveal that treating residual value as a static scalar tied to current state-of-health is insufficient for decentralized community operation. Instead, value evolves jointly with remaining useful life and scenario adaptability, and intensive dispatch strategies impose quantifiable degradation burdens that erode this value over time. Incorporating both the dynamic residual-value signal and the operational degradation burden into the game-theoretic payoff structure allows the community operator and service provider to internalize long-term battery-health costs rather than optimizing solely for short-term dispatch revenue. This reallocation of economic attention—from immediate energy arbitrage to life cycle value preservation—is consistent with the core circular-economy principle that asset longevity, not merely throughput, should guide resource coordination.

At the community level, the Stackelberg equilibrium results indicate that subsidy design must be sensitive to battery health heterogeneity. Uniform allocation fails to capture the reality that healthier retired batteries generate higher welfare, stronger utilization, and lower required compensation, whereas degraded batteries demand more careful incentive calibration to remain economically viable. The proposed dynamic redistribution mechanism, which weights subsidies by degradation contribution, participation duration, and circular-economy correction factors, consistently outperforms uniform allocation across subsidy intensities and perturbation scenarios. This suggests that the effectiveness of community storage incentives depends less on aggregate subsidy magnitude than on the microstructure of allocation rules. From a policy perspective, the finding that welfare peaks at moderate subsidy intensity and declines under excessive uniform subsidies carries direct implications for subsidy budget design: targeted, condition-responsive allocation is preferable to scale expansion alone.

The sensitivity and robustness analyses further show that the mechanism remains stable when residual-value mapping weights, circular-economy correction factors, or degradation thresholds are moderately perturbed. Welfare variation stays within narrow bounds across parameter settings, and the mechanism maintains superior median and worst-case performance under sample anomaly, price disturbance, participant withdrawal, and load anomaly. This stability is practically important because decentralized communities operate with incomplete information and stochastic participation; an incentive mechanism that collapses under minor perturbations would be unsuitable for real-world deployment. The acceptable computational cost for solving the reformulated mixed-integer equilibrium additionally supports the feasibility of day-ahead or rolling-horizon community scheduling.

### Limitations of the study

Several limitations should be acknowledged. First, the battery-aging dataset, while covering LFP (Lithium Iron Phosphate), NMC, and NCA chemistries, does not yet include emerging systems such as solid-state or sodium-ion batteries, and the community scenario is based on a typical residential setting rather than industrial or commercial complexes. Second, the game structure assumes cooperative signal setting between the operator and the service provider; strategic conflicts or information asymmetry at the upper level are not modeled. Third, the scenario-adaptability weights and circular-economy correction factors are currently preset; their data-driven optimization remains for future work. Future research should extend the degradation-prediction framework to broader chemistries and richer field datasets; develop multi-level multi-timescale game structures that capture interactions across electricity markets, communities, and service providers; and refine the circular-economy value framework through reinforcement learning or multi-objective optimization to yield more rigorous, measurable allocation weights. These extensions would deepen the integration of decentralized energy communities with retired-battery value chains and move closer to a genuinely closed-loop circular economy for energy storage.

## Resource availability

### Lead contact

Requests for further information and resources should be directed to and will be fulfilled by the lead contact, Yashu Chen (2003108@aust.edu.cn).

### Materials availability

This study did not generate new unique reagents.

### Data and code availability

• This study uses publicly available battery-aging datasets from the MATR Data Platform (https://data.matr.io/1/projects/5c48dd2bc625d700019f3204) and Mendeley Data: https://doi.org/10.17632/nsc7hnsg4s.2. The data collected from the 170 commercial retired battery modules measured by the authors are available from the lead contact upon reasonable request to reanalyze the data reported in this paper.
• All original code supporting this study is publicly available via OSF at https://doi.org/10.17605/OSF.IO/KPU29.
• Any additional information required to reanalyze the data reported in this paper is available from the lead contact upon request.

## Acknowledgments

This work was supported by Anhui Provincial Social Science Innovation and Development Research Project: Reshaping the Competitiveness of Resource-Based Cities in Anhui Province from the Perspective of Innovation Drive (grant no. 2024CX069) and Anhui Higher Educational Project of Excellent Scientiffc Research and Innovation Team (grant no. 2023AH010026).

## Author contributions

G.L. performed conceptualization, methodology, model design, experiments, writing – original draft, and revision. Y.C. conducted data curation, formal analysis, visualization, and writing – review and editing. D.Y. provided supervision, project administration, funding acquisition, and writing – review and editing. J.W. provided writing – review and editing.

## Declaration of interests

The authors declare no competing interests.

## STAR★Methods

### Key resources table

**Table:** 

REAGENT or RESOURCE	SOURCE	IDENTIFIER
**Software and algorithms**		

Python (programming language, v3.9)	Python Software Foundation	https://www.python.org/
Pyomo (optimization modeling language)	Sandia National Laboratories	https://www.pyomo.org/
Gurobi Optimizer (v11.0)	Gurobi Optimization, LLC	https://www.gurobi.com/
Physics-constrained transfer-learning hybrid capacity-degradation prediction model	This paper	https://doi.org/10.17605/OSF.IO/KPU29
Stackelberg game equilibrium solver (KKT reformulation and mixed-integer optimization)	This paper	https://doi.org/10.17605/OSF.IO/KPU29

**Other**		

Retired LFP battery modules (18650, 21700, and pouch formats; SOH 50%–82%)	This paper	N/A
Residential community operation dataset ( …)	This paper	N/A
Intel Core i7 processor workstation	N/A	N/A
NVIDIA GeForce RTX 3080 GPU	NVIDIA Corporation	N/A

### Experimental model and study participant details

This study does not involve human participants, animals, cell lines, or other biological materials. The experimental framework comprises a mixed-source lithium-ion battery aging dataset and a numerical residential energy-community model. The battery data were used to train and evaluate the capacity-degradation prediction model, whereas the community-operation data were used to construct and analyze the game-theoretic energy-management scenario.

#### Battery aging dataset

The battery-aging dataset comprised 240 samples collected from two sources. Among these, 170 samples were obtained from commercial retired lithium iron phosphate (LFP), nickel manganese cobalt (NMC), and nickel cobalt aluminum (NCA) battery modules independently tested by the authors. The remaining 70 samples were selected from publicly available lithium-ion battery cycling and aging datasets reported by Severson et al.^36^ and Ma et al.^37^ The author-measured samples and public samples accounted for 70.8% and 29.2% of the complete dataset, respectively.

The dataset included 18650 cylindrical cells, 21700 cylindrical cells, and pouch-format batteries. The samples covered state-of-health (SOH) values ranging from 50% to 82%, cycling temperatures primarily between 20°C and 35°C, and discharge rates ranging from 0.25C to 1C. These samples were pooled to evaluate capacity-degradation prediction under heterogeneous battery chemistries, formats, aging states, and operating conditions.

#### Battery data preprocessing

The author-measured and publicly available samples were processed using a unified calibration and normalization procedure before model training and evaluation. Differences in rated capacity and nominal cycle life were normalized to improve comparability across battery types. SOH was calculated using a consistent capacity-based definition, and remaining useful life (RUL) was recalibrated according to the common second-life end-of-life threshold adopted in this study. The resulting mixed-source dataset was used for model training, validation, and performance evaluation.

#### Residential energy-community scenario

The community-operation dataset was constructed from operating records and configuration parameters for a typical small residential community. It included rooftop photovoltaic generation, household electricity demand, time-of-use electricity prices, and retired-battery storage-dispatch information at a 15-min temporal resolution over one year. The numerical community contained 60 prosumers, a total photovoltaic capacity of 245 kWp, and a shared retired-battery storage system with a capacity of 380 kWh.

These data were used exclusively to construct the numerical energy-community scenario and were not included in the training of the battery-degradation prediction model. The scenario was used to evaluate Stackelberg equilibrium outcomes, residual-value-oriented storage operation, subsidy allocation, community welfare, and robustness under different battery and operational conditions.

#### Baseline parameter settings

Unless otherwise specified, the circular-economy correction weights were set to η_1_ = η_2_ = η_3_ = 1/3. This equal-weight setting represents a neutral baseline in which resource-recovery and conservation contribution, carbon-emission-reduction contribution, and community-stability contribution are treated as equally important. Parameters without directly measured values were specified as scenario-based baseline settings and were subsequently examined through sensitivity and robustness analyses.

#### Computational environment and reproducibility

The capacity-degradation prediction model and community simulations were implemented in Python. The Stackelberg game was formulated in Pyomo, and the Karush–Kuhn–Tucker-reformulated mixed-integer optimization problem was solved using Gurobi 11.0. All numerical experiments were conducted on a workstation equipped with an Intel Core i7 processor and an NVIDIA GeForce RTX 3080 GPU. Random seeds were fixed from 1 to 10 for repeated stochastic experiments, and the results were reported as the mean and standard deviation across the corresponding runs.

### Method details

#### Overall framework

The proposed framework integrates capacity-degradation prediction, residual-value assessment, Stackelberg game coordination, and dynamic subsidy allocation. Predicted capacity trajectories and remaining useful life (RUL) are converted into residual-value and degradation-burden signals, which are incorporated into community operation and subsidy decisions. The overall framework is shown in Figure 1.

#### Capacity-degradation prediction

##### Feature extraction and physics-constrained representation

For each cycle, voltage, current, temperature, and state-of-charge measurements are processed through three parallel branches that extract waveform, statistical, and incremental-capacity features. These features are fused with a semi-empirical degradation descriptor accounting for cycling aging, calendar aging, temperature acceleration, and operating stress^38^^,^^39^:

(Equation 1) $$Q_{k}^{\text{phy}}=Q_{0}\cdot \left[1-\alpha \cdot N_{k}^{\beta }-\gamma \cdot \int_{0}^{t_{k}}f\left(T,\text{SOC},I\right)\;d\tau \right]$$

(Equation 2) $$f\left(T,\text{SOC},I\right)=\exp\left(-\frac{E_{a}}{RT}\right)g\left(\text{SOC}\right)\cdot {\left|I\right|}^{\theta }$$

The fused representation preserves data-driven degradation characteristics while introducing physically meaningful constraints. The complete model architecture is shown in Figure 2, and the physical-prior and training parameters are summarized in Table 1.

#### Cross-chemistry alignment

To improve transferability across heterogeneous battery chemistries, covariance alignment is applied to the physics-constrained latent representations. In the representative transfer task, NMC batteries constitute the source domain and NCA batteries constitute the target domain:

(Equation 3) $$L_{\text{align}}=\frac{1}{4d^{2}}∥C_{\text{NMC}}-C_{\text{NCA}}{∥}_{F}^{2}$$

This constraint reduces latent distribution differences while retaining chemistry-dependent degradation information.

#### Temporal prediction and training

The aligned features are processed by a Bi-LSTM with an attention mechanism to predict the capacity trajectory. The total training objective combines prediction error, physics-consistency regularization, monotonicity regularization, and cross-chemistry alignment:

(Equation 4) $$L_{\text{phy}}=\frac{1}{N}\sum_{k=1}^{N}{\left({\hat{Q}}_{k}-Q_{k}^{\text{phy}}\right)}^{2}$$

(Equation 5) $$L_{\text{mono}}=\frac{1}{N-1}\sum_{k=1}^{N-1}\max\;{\left(0,{\hat{Q}}_{k+1}-{\hat{Q}}_{k}\right)}^{2}$$

(Equation 6) $$L=\frac{1}{N}\sum_{k=1}^{N}{\left({\hat{Q}}_{k}-Q_{k}\right)}^{2}+\lambda_{1}L_{\text{phy}}+\lambda_{2}L_{\text{mono}}+\lambda_{3}L_{\text{align}}$$

The physics-consistency term constrains predictions toward the semi-empirical degradation trend, whereas the monotonicity term penalizes physically unreasonable capacity increases. RUL is determined from the predicted cycle at which capacity reaches the second-life end-of-life threshold, set to (Q_th_ = 0.5Q_0_).

#### Residual-value assessment

##### Dynamic residual-value mapping

The residual value of a retired battery is determined by residual capacity, predicted RUL, and application-scenario adaptability:

(Equation 7) $$V_{t}^{\text{res}}=\omega_{1}\cdot \frac{Q_{t}}{Q_{0}}\cdot V_{0}^{\text{cap}}+\omega_{2}\cdot \frac{{\text{RUL}}_{t}}{{\text{RUL}}_{0}}\cdot V_{0}^{\text{life}}+\omega_{3}\cdot \psi \left(\text{App}\right)$$

The capacity-value and lifespan-value benchmarks were normalized to 1 because the analysis focuses on relative value evolution. Scenario adaptability incorporates capacity matching, power-rate compatibility, cycle-depth suitability, safety margin, and operational stability. Values of 0.60, 0.75, and 0.90 represent low, medium, and high suitability for residential community storage, respectively.

#### Degradation burden

The degradation burden represents the cumulative capacity loss induced by the charging and discharging schedule:

(Equation 8) $$\mathrm{Deg}\left(p\right)=\sum_{t\in T}\Delta Q_{t}\left(p\right)$$

The residual-value signal enters the service provider’s payoff function, while the degradation burden enters the community operator’s objective as an operating cost.

#### Stackelberg game formulation

##### Participants and objectives

The community operator and retired-battery service provider form the upper-level leader coalition, whereas prosumers act as lower-level followers. The operator determines electricity prices, subsidies, and storage dispatch. The service provider determines battery availability and service utilization according to residual value, maintenance cost, and degradation loss. Prosumers respond through electricity transactions, flexible consumption, photovoltaic self-consumption, and storage participation. Their strategic relationships are shown in Figure 3.

The operator maximizes storage-dispatch revenue after operating and degradation costs. Prosumers minimize net energy costs after accounting for electricity transactions, consumption adjustments, and received subsidies.

#### Equilibrium solution

The leader coalition announces its pricing, subsidy, dispatch, and utilization decisions, after which prosumers determine their optimal responses. The prosumer objective is convex, and its feasible region is assumed to be nonempty, closed, bounded, and convex. The lower-level optimum can therefore be represented using the Karush–Kuhn–Tucker conditions:

(Equation 9) $$\begin{array}{l} \nabla_{q_{i}}L_{i}=0\\ g_{i,m}\left(q_{i}\right)\leq 0,h_{i,r}\left(q_{i}\right)=0\\ \alpha_{i,m}\geq 0\\ \alpha_{i,m}g_{i,m}\left(q_{i}\right)=0,\forall m\in M_{i} \end{array}$$

The complementary-slackness conditions are linearized using binary variables and a sufficiently large Big-M constant, yielding a single-level mixed-integer optimization model.

#### Dynamic subsidy allocation

##### Degradation-sensitive contribution

A threshold-constrained response function distinguishes normal storage participation from degradation-intensive operation:

(Equation 10) $$\varphi \left(\Delta Q_{t}\right)=\left\{ \begin{array}{l} \Delta Q_{t},0\leq \Delta Q_{t}\leq \Delta Q^{th}\\ \Delta Q^{th}\;\exp\left[-\eta_{d}\frac{\Delta Q_{t}-\Delta Q^{th}}{\Delta Q^{th}}\right],\Delta Q_{t}>\Delta Q^{th} \end{array}\right.$$

Below the allowable degradation threshold, the contribution weight increases with normal battery use. Above the threshold, exponential attenuation reduces the weight to prevent excessive degradation from generating additional subsidies.

The effective contribution of each prosumer combines degradation response, active participation, and circular-economy performance:

(Equation 11) $${\text{Cont}}_{i}=\sum_{t\in T}\frac{\varphi \left(\Delta Q_{t}\right)\cdot 1\left\{i\in A_{t}\right\}}{\left|A_{t}\right|+\varepsilon }\cdot \xi_{i}$$

#### Circular-economy correction and subsidy distribution

The circular-economy correction factor combines normalized resource-conservation, carbon-emission-reduction, and community-stability contributions^40^^,^^41^:

[Insert the circular-economy correction factor as Equation 11.]

(Equation 12) $$\begin{array}{l} \xi_{i}=1+\eta_{1}{\tilde{R}}_{i}+\eta_{2}{\tilde{C}}_{i}+\eta_{3}{\tilde{S}}_{i}\\ \eta_{1}+\eta_{2}+\eta_{3}=1,\eta_{1},\eta_{2},\eta_{3}\geq 0 \end{array}$$

The baseline setting uses equal weights, (η_1_ = η_2_ = η_3_ = 1/3). Alternative policy preferences are evaluated in the sensitivity analysis.

The equilibrium subsidy is redistributed according to each prosumer’s effective contribution and participation duration:

(Equation 13) $$s_{i,t}=\zeta_{t}^{*}\cdot \frac{{\text{Cont}}_{i}\cdot \left(1-e^{-\delta \cdot \tau_{i}}\right)}{\sum_{j\in N}{\text{Cont}}_{j}\cdot \left(1-e^{-\delta \cdot \tau_{j}}\right)}$$

This allocation rule rewards useful and sustained participation while accounting for battery degradation and circular-economy benefits.

### Quantification and statistical analysis

#### Experimental setup

The battery-degradation experiments used 240 aging samples, including 170 retired LFP, NMC, and NCA battery modules measured by the authors and 70 samples from public datasets. All samples underwent unified capacity normalization, SOH calculation, and RUL recalibration before analysis. Community-level simulations considered 60 prosumers, 245 kWp of photovoltaic capacity, 380 kWh of retired-battery storage, and 96 dispatch intervals per day. The models were implemented in Python and Pyomo, and the KKT-reformulated optimization problem was solved using Gurobi 11.0.

#### Performance metrics

Capacity-degradation prediction was evaluated using root-mean-square error (RMSE), mean absolute error (MAE), and the coefficient of determination (R^2^). Residual-value consistency was assessed using RMSE and MAE between values derived from predicted and measured degradation trajectories. Community-level performance was evaluated using operator revenue, prosumer savings, service-provider revenue, storage utilization, self-consumption ratio, subsidy expenditure, and total community welfare.

#### Comparative and ablation analyses

The proposed prediction model was compared with LSTM, GRU with attention, Bi-LSTM, CNN-Bi-LSTM, and a physics-informed neural network. Cross-chemistry generalization was assessed under direct transfer, raw-feature maximum mean discrepancy alignment, and the proposed physics-constrained latent alignment. Ablation experiments separately removed the physics-consistency loss, monotonicity constraint, cross-chemistry alignment, incremental-capacity feature branch, and attention head while retaining the remaining model configuration.

#### Sensitivity and robustness analyses

Sensitivity analyses varied the residual-value weights, circular-economy correction weights, degradation threshold, penalty coefficient, and subsidy intensity. Robustness was further evaluated under battery-sample anomalies, electricity-price disturbances, participant withdrawal, and load anomalies. Median welfare, interquartile range, worst 5% welfare quantile, and solution time were used to characterize operational stability and downside performance.

#### Statistical reporting

All stochastic prediction experiments were repeated using random seeds 1–10, and the results are reported as mean ± standard deviation. Community-operation results are reported in their original monetary or Normalized units. Robustness outcomes are summarized using distributional statistics rather than relying only on mean values. No formal null-hypothesis significance testing was applied.

#### Data and software availability

The public battery-aging datasets used in this study are available from the MATR Data Platform (https://data.matr.io/1/projects/5c48dd2bc625d700019f3204) and Mendeley Data (https://doi.org/10.17632/nsc7hnsg4s.2). The data collected from the 170 commercial retired battery modules measured by the authors are available from the lead contact upon reasonable request to reanalyze the data reported in this paper. All original code supporting this study, including the physics-constrained transfer-learning hybrid prediction model and the Stackelberg game equilibrium solver, is publicly available via OSF at https://doi.org/10.17605/OSF.IO/KPU29. Any additional information required to reanalyze the data reported in this paper is available from the lead contact upon request.

### Additional resources

No additional external resources were generated in this study.
